# Female predominance and socio-demographic inequalities in global near vision loss burden: projected trends and disparities from 1990 to 2035

**DOI:** 10.3389/fpubh.2025.1611433

**Published:** 2025-11-03

**Authors:** Guodong Tang, Jing Li, Xiaoqi Gong, Han Yu, Man Jiang, Yibo Han, Dongfang Wang, Yuxi Liu, Jike Song, Hongsheng Bi

**Affiliations:** ^1^Affiliated Eye Hospital of Shandong University of Traditional Chinese Medicine, Jinan, China; ^2^Shandong University of Traditional Chinese Medicine, Jinan, China; ^3^Shandong Provincial Key Laboratory of Integrated Traditional Chinese and Western Medicine for Prevention and Therapy of Ocular Diseases, Shandong Academy of Eye Disease Prevention and Therapy, Jinan, Shandong, China

**Keywords:** near vision loss, Global Burden of Disease, health disparities, aging population, socioeconomic determinants

## Abstract

**Background:**

Near vision loss (NVL), a hallmark of aging populations, imposes a growing global health burden, exacerbated by demographic shifts and socioeconomic disparities. Despite its profound impact on productivity and quality of life, comprehensive analyses that integrate aging, socioeconomic development, and sex-specific disparities remain limited.

**Methods:**

Using the Global Burden of Disease (GBD) 2021 dataset spanning 204 countries and territories (1990–2021), we evaluated the NVL burden through prevalence, disability-adjusted life years (DALYs), and sociodemographic index (SDI). Advanced methodologies included decomposition analysis to disentangle demographic and epidemiological drivers, Bayesian age-period-cohort (BAPC) modeling for projections, and frontier analysis to quantify SDI-linked inequities. Gender-stratified and age-specific trends were analyzed using joinpoint regression analysis.

**Results:**

Global NVL prevalence surged from 428 million to 1.155 billion cases (1990–2021). Females exhibited a higher age-standardized prevalence (16,588 vs. 14,718 per 100,000 in 2021). South Asia had the highest burden (age-standardized DALYs: 208.0), whereas the Gulf Cooperation Council reported the lowest (93.8). Socioeconomic inequities widened: The DALY gap between high- and low-SDI regions expanded from 10.19 to 31.96. Population growth (65.3%) and epidemiological shifts (39.2%) drove DALY increases, offset marginally by aging (−4.4%).

**Conclusion:**

NVL burden escalated disproportionately in low-SDI regions and among females, fueled by population growth and systemic healthcare gaps. Aging, while a minor contributor globally, critically affects the high-income Asia-Pacific region. Policymakers must prioritize sex-sensitive refractive care programs, expand optical subsidies in underserved areas, and address digital near-work hazards to mitigate the premature onset of NVL.

## 1 Introduction

According to the World Health Organization, between 2015 and 2050, the proportion of the global population aged over 60 years will nearly double (from 12 to 22%) (1). This unprecedented demographic shift means that age-related conditions, such as presbyopia, will affect an ever-growing number of people in the coming decades. Within the Global Burden of Disease (GBD) framework, near vision loss (NVL) denotes near-vision impairment attributable to uncorrected presbyopia, whereas presbyopia refers to age-related loss of accommodation. This phenomenon can be attributed to age-related stiffening of the lens, which impedes optimal near vision (2). Current estimates indicate that this condition affects more than 1.8 billion individuals worldwide (3). Individuals with uncorrected NVL frequently encounter challenges while performing close-up tasks. Research indicates that 50%–70% of individuals with functional presbyopia experience difficulties with near work, resulting in absenteeism, reduced work efficiency, and a decline in economic productivity (4–6). From an economic standpoint, the absence of treatment for presbyopia significantly contributes to productivity losses, with annual vision-related expenditures of $25.4 billion (7). More recent analyses focusing on low- and middle-income countries and territories (where the majority of NVL cases reside) found more than $54.1 billion per year in productivity loss in these regions alone due to uncorrected presbyopia (8). Evidence from qualitative studies and limited quantitative research indicates that presbyopia exerts a substantial impact on quality of life across domains such as activity limitations, emotional well-being, social status, and economic standing—an effect that is frequently underestimated (9–13).

Interventions that correct presbyopia—including optical correction (spectacles, contact lenses), surgical approaches (corneal or lens-based procedures), and pharmacological therapies—can reduce the burden of NVL (14, 15). Presbyopia-related near-vision impairment can be effectively corrected with low-cost optical interventions (e.g., ready-made or custom near spectacles, bifocal/multifocal designs), and widespread correction substantially reduces population-level NVL; however, unmet needs remain considerable (16). In low-income regions, only 6%–45% of individuals requiring correction receive adequate care (17, 18). The highest reported correction rates were observed in Los Angeles, USA (87.4%) (19), followed by Trinidad and Tobago (52.6%) (20), and Parintins, Brazil (31.4%) (21, 22). A randomized trial in rural India demonstrated that tea pickers aged ≥40 years with corrected NVL achieved a 22% increase in productivity (and income growth) compared with their uncorrected counterparts (23). These findings highlight the urgency of focusing on NVL, which should not be viewed merely as a biological inevitability but as a modifiable determinant of health equity.

This study aimed to systematically address critical gaps in understanding the global burden of NVL by integrating socioeconomic development, demographic aging, and health policy dimensions. While previous studies have inadequately disentangled the contributions of population growth, aging, and epidemiological shifts to the NVL burden or accounted for regional disparities in intervention efficacy, our analysis leveraged the GBD 2021 dataset across 204 countries and territories to fill these voids. Employing advanced methodologies, including decomposition analysis to isolate demographic and epidemiological drivers, Bayesian age-period-cohort (BAPC) models for granular future projections, and frontier analysis to quantify sociodemographic index (SDI)-linked inequities, we mapped the evolving burden from 1990 to 2021 and identified context-specific mitigation strategies. This study resolves the limitations of fragmented regional assessments and simplistic forecasting, thereby providing a comprehensive framework for prioritizing interventions—such as optical subsidy expansion and workplace vision policy reforms—particularly in underserved low-resource settings where diagnostic and care gaps persist.

## 2 Materials and methods

### 2.1 Data source

We conducted a retrospective study using population data from the Global Health Data Exchange database. This dataset provides a detailed analysis of 371 diseases and injuries, with a particular focus on NVL, covering the period from 1990 to 2021 across the GBD super regions and 204 countries and territories. The methods used in the GBD 2021 have been extensively documented in previous studies. NVL-related data, accessed via the Global Health Data Exchange,^1^ include: (1) Global trends: total prevalence and disability-adjusted life years (DALYs) (1990–2021) reported as absolute numbers and age-standardized rates (per 100,000 population) to control for demographic differences, with stratification by age and gender; (2) Regional and national patterns: Prevalence and DALYs across GBD super regions and 204 countries and territories (1990–2021), with absolute counts and age-standardized rates for cross-regional comparisons; and (3) Socioeconomic context: SDI values for 1990–2021, reflecting national development levels, calculated by combining the total fertility rate under age 25, mean years of schooling for those aged 15 and older, and lag-distributed income per capita (24). This index, calculated at the national level, accurately reflects the level of social development across countries and territories. Ethical approval and informed consent were not required for this study.

### 2.2 Case definition

In line with the GBD framework, near vision loss (NVL) denotes uncorrected presbyopia, defined as presenting near vision worse than N6 or N8 at 40 cm in individuals with best-corrected distance visual acuity >6/12; estimates are derived from measured acuity, and self-reported data are excluded.

### 2.3 Statistics

The DALYs metrics, comprising rates and case counts, form the basis for evaluating DALY-related burdens. Rates are expressed per 100,000 population estimates, whereas case counts represent the absolute burden derived from the total instances. Both measures are reported at 95% uncertainty intervals (UI). Statistical evaluations were conducted using suitable models with a significance level of *p* < 0.05. Techniques such as the slope index of inequality, concentration index, frontier and decomposition analyses, joinpoint regression, and the BAPC model are described in subsequent sections. The World Health Organization Health Equity Assessment Toolkit and R software (v4.3.2) facilitated all computations and visualizations. All statistical analysis R scripts are available in Supplementary materials to ensure transparency and reproducibility.

Joinpoint regression was employed to determine trends in disease burden and evaluate temporal patterns in NVL between 1990 and 2021. This approach employs piecewise regression under a log-linear framework, represented by ln(y)=β·x+constant , to identify change points. The grid search method (GSM) was used to locate potential join points, choosing the model with the minimum mean squared error (MSE) as optimal. A Monte Carlo permutation test was applied to ascertain the ideal number of join points, permitting up to five points and as few as zero. The final model computed the annual percentage change (APC), average annual percentage change (AAPC), estimated annual percentage change (EAPC), and 95% confidence intervals (CI) globally and across SDI regions, assessing trend variations over the study period. APC was derived from $\mathrm{APC}=(e^{\beta }-1)\times 100\%$ , where β denotes the log-linear regression coefficient. The AAPC serves as a composite measure, aggregating the APCs across temporal segments to reflect overarching trends. Furthermore, the EAPC is a significant indicator for evaluating trends, and the methodology for its calculation has been documented previously (25).

### 2.4 Cross-country inequality analysis

This analysis used the World Health Organization’s slope index of inequality (SII) and concentration index to evaluate the absolute and relative disparities in the burden of refractive disorders and NVL across nations and regions. The SII quantifies inequality by modeling DALY rates against the midpoints of the cumulative population distributions ranked by the SDI. For temporal comparisons of health disparities, data spanning 204 countries and territories from 1990 to 2021 were analyzed. A robust regression model (RLM) was prioritized over ordinary linear regression (LM) to mitigate bias and heterogeneity. The concentration index, calculated by contrasting the cumulative DALY proportions with the population distributions ordered by the SDI, integrates the area under the Lorenz curve to measure inequity.

### 2.5 Frontier analysis and decomposition analysis

For each year we constructed a monotone empirical frontier of the lowest attainable DALY rate at a given SDI. Specifically, within each of B = 100 bootstrap samples (resampling countries with replacement), countries were sorted by SDI and the running minimum (cummin) of the age-standardized DALY rate was taken to define a non-increasing frontier along the SDI axis. The average frontier across bootstrap replicates was used for estimation; a LOESS smooth (span = 0.2) was superimposed for visualization only. The efficiency gap for a country-year was defined as observed ASR minus the frontier ASR at the same SDI. Complementing this, the Das–Gupta decomposition method parsed NVL burden trends (1990–2021) into contributions from aging, population shifts, and epidemiological transitions. This granular evaluation disentangles the drivers of temporal patterns, offering insights that are distinct from linear regression variable correlations.

### 2.6 BAPC model projection

We projected global DALY counts to 2035 using BAPC with INLA. Observed counts per 5-year age group (1990–2021) were arranged in an age×year matrix; population counts provided Poisson offsets (log-link). We formed data-driven standard weights as the mean age shares across 1990–2021 and used them to compute posterior age-standardized rates. The linear predictor decomposed into age, period, and cohort effects with the following priors: age ~ RW2, period ~ RW1, cohort ~ RW2, and an IID over-dispersion term; hyperpriors followed log-Gamma settings used in the scripts (age/period/cohort precision log-Gamma(1, 0.00005); over-dispersion log-Gamma(1, 0.005)). We set secondDiff = FALSE. Forecasts used npredict = 2035–2021, with retro = TRUE. Posterior means and approximate 95% credible intervals for ASR (per 100,000) were plotted, with the projection period (2022–2035) highlighted distinctly. Assumptions included stable case definitions, smooth continuation of APC effects in the absence of shocks, and demographic trajectories consistent with GBD population forecasts.

## 3 Results

### 3.1 Global burden and trends in NVL

Globally, the burden of NVL increased from 1990 to 2021. Prevalent cases rose from 428.0 million (95% UI: 323.0–562.0 million) to 1.155 billion (95% UI: 0.875–1.515 billion). The age-standardized prevalence rate (ASPR) increased with an EAPC of 1.47 (95% CI: 1.31–1.63). DALYs increased from 4.316 million (95% UI: 1.937–8.411 million) to 11.650 million (95% UI: 5.214–22.422 million), and the EAPC for the age-standardized DALY rate was 1.50 (95% CI: 1.33–1.66) (Table 1).

**Table 1:** Global, sex-specific, and SDI region–specific NVL prevalence and DALY metrics in 1990 and 2021, including the number of cases, age-standardized rates (per 100,000 population), and the estimated annual percentage change (EAPC) from 1990 to 2021.

Category	Prevalence					DALYs				
	Number of cases, 1990	Age-standardized rate per 100,000 population, 1990	Number of cases, 2021	Age-standardized rate per 100,000 population, 2021	Estimated annual percentage change, 1990–2021	Number of cases, 1990	Age-standardized rate per 100,000 population, 1990	Number of cases, 2021	Age-standardized rate per 100,000 population, 2021	Estimated annual percentage change, 1990–2021
Global	427937731.057 (322776201.962–561645080.040)	9788.658 (7371.641–12888.266)	1155063049.587 (875226145.904–1514597489.373)	13436.185 (10223.449–17585.835)	1.47 (1.31,1.63)	4316104.573 (1937499.865–8410596.300)	98.261 (44.110–190.988)	11649944.750 (5214018.649–22421658.631)	135.518 (60.823–260.389)	1.50 (1.33,1.66)
Sex										
Male	184798761.444 (137901354.301–245460367.836)	8753.843 (6579.747–11516.202)	512274961.077 (381265146.947–680315158.670)	12207.945 (9158.600–16145.295)	1.52 (1.36,1.69)	1875879.501 (833571.950–3699968.435)	88.236 (39.453–171.318)	5201906.934 (2330305.031–9943701.591)	123.787 (55.554–236.594)	1.55 (1.38,1.72)
Female	243138969.613 (183232395.741–316953903.954)	10765.233 (8127.189–14148.518)	642788088.510 (494003521.829–833474438.999)	14615.504 (11218.522–18924.042)	1.44 (1.28,1.59)	2440225.071 (1096944.886–4726477.791)	107.756 (48.473–209.574)	6448037.817 (2884324.542–12279477.274)	146.820 (65.622–279.717)	1.46 (1.30,1.62)
SDI region										
Middle SDI	136883044.500 (103329965.057–177666072.725)	11085.312 (8446.412–14432.870)	429839886.238 (322949503.744–565578250.526)	15372.700 (11707.711–20094.478)	1.49 (1.33,1.66)	1387021.690 (616293.982–2687935.631)	111.267 (49.936–215.038)	4344198.612 (1940326.596–8347958.798)	155.092 (69.329–297.136)	1.52 (1.35,1.69)
Low SDI	37046595.940 (27880465.606–49056856.527)	12448.641 (9373.220–16427.886)	104595573.534 (79346300.033–136002120.639)	16252.924 (12370.249–20760.610)	1.48 (1.27,1.70)	373195.633 (165509.615–728169.028)	126.396 (56.290–247.065)	1059106.842 (468419.577–1992365.694)	152.080 (67.799–285.900)	1.05 (0.90,1.19)
High SDI	92236873.557 (68939826.728–122669436.777)	6386.770 (4701.059–8555.866)	268439314.225 (202599984.016–348791237.958)	6970.864 (5146.039–9289.419)	0.48 (0.41,0.54)	965522.945 (424555.523–1874373.435)	93.462 (41.322–181.121)	2383039.968 (1087866.741–4527647.124)	129.039 (58.411–248.065)	1.44 (1.27,1.60)
High-middle SDI	95901983.547 (72245029.183–126357813.912)	12691.030 (9558.956–16621.861)	236295922.807 (175829654.001–310139117.829)	15178.137 (11707.316–19284.757)	1.01 (0.87,1.16)	658922.544 (297976.635–1297099.925)	64.409 (28.767–126.816)	1153259.188 (522018.132–2212992.684)	70.307 (31.424–135.993)	0.48 (0.41,0.54)
Low-middle SDI	65507368.481 (47979250.180–87725743.847)	9312.153 (7060.474–12156.859)	115266965.773 (85334195.269–152976369.782)	12763.980 (9620.796–16797.590)	1.40 (1.24,1.57)	927805.507 (419576.853–1820428.590)	123.992 (55.317–239.797)	2704079.146 (1209008.362–5150525.804)	162.795 (72.895–306.385)	1.52 (1.30,1.74)

**Figure 1:**
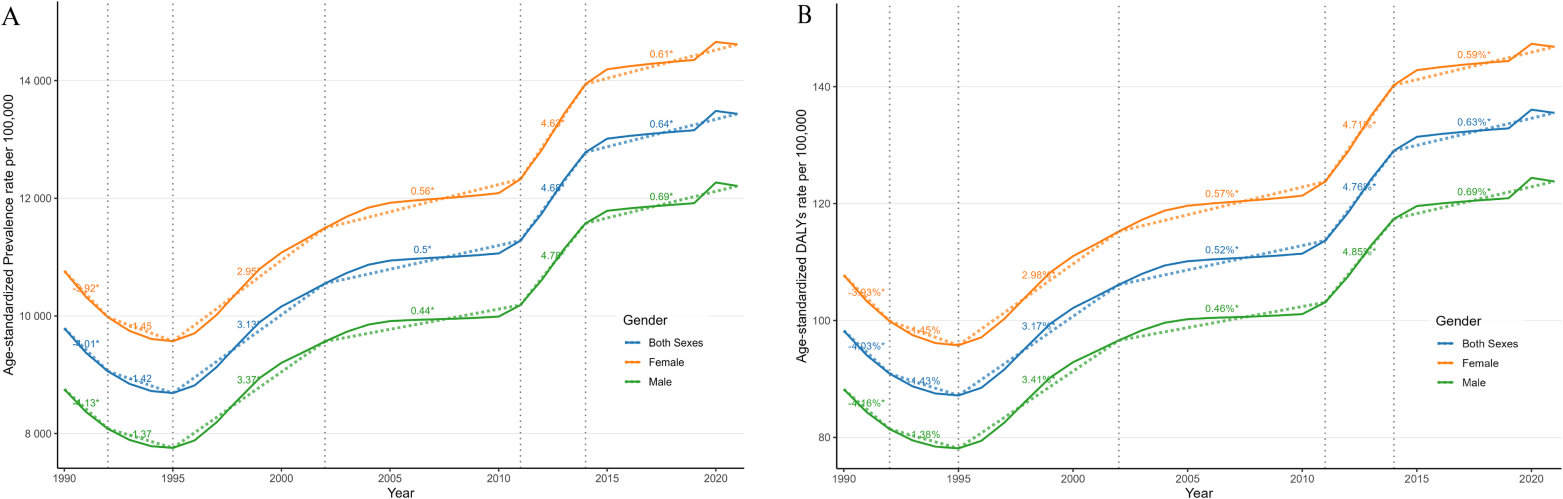
Joinpoint regression analysis of the global prevalence of NVL **(A)** and NVL DALYs **(B)** from 1990 to 2021. APC, annual percentage change; DALYs, disability-adjusted life years.

Gender-specific analysis revealed that males exhibited slightly higher growth rates in both ASPR (EAPC = 1.52) and age-standardized DALYs (EAPC = 1.55) than females (1.44 and 1.46, respectively). However, females consistently demonstrated higher ASPR and age-standardized DALYs than males over the 30-year study period (Table 1 and Figures 1A,B). Segmented APC analysis indicated that ASPR and age-standardized DALYs experienced a brief decline from 1990 to 1992, reached peak growth rates from 1995 to 2002, showed a resurgence from 2011 to 2014, followed by a deceleration in growth.

**Figure 2:**
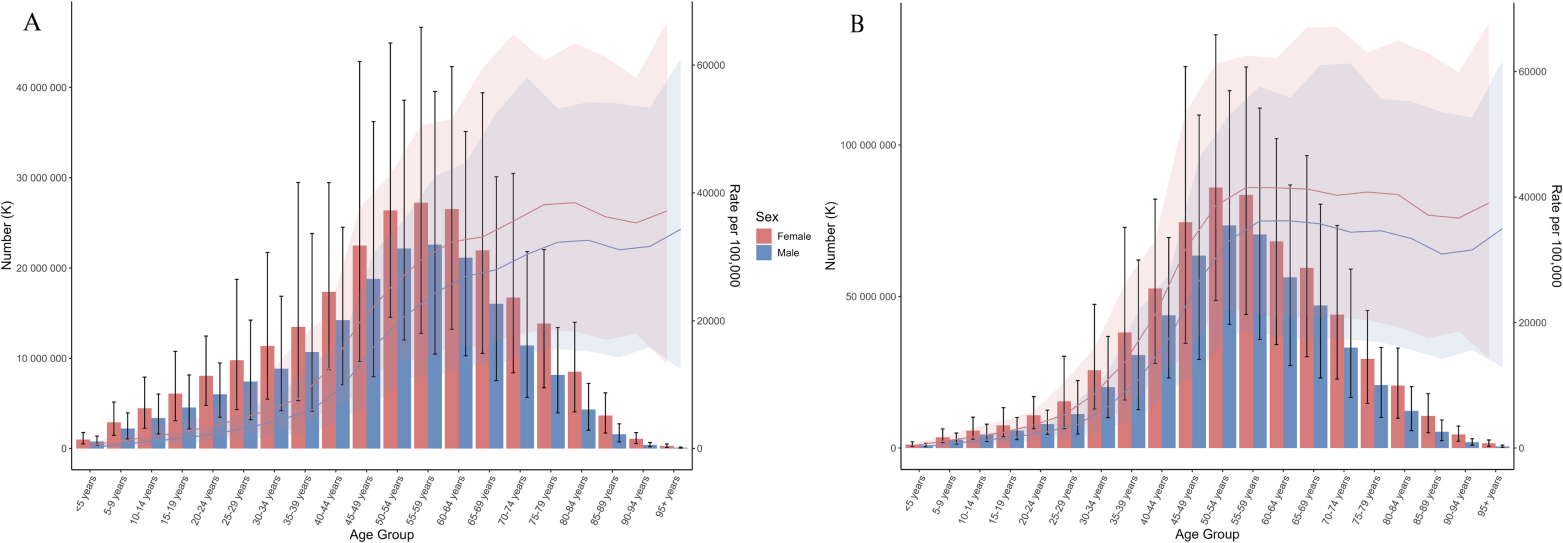
Age-specific rate and number for prevalence of NVL between 1990 **(A)** and 2021 **(B).**

In 1990, the highest number of prevalent cases was observed in the 55–59 age group, with 27.24 million (95% CI: 12.77 million to 46.65 million) for females and 22.60 million (95% CI: 10.47 million to 39.55 million) for males. The peak prevalence number shifted to the 50–54 age group by 2021, reaching 73.46 million (95% CI: 40.85 million to 117.90 million) for females and 86.01 million (95% CI: 48.72 million to 136.35 million) for males (Figures 2A,B and Supplementary Table S1).

### 3.2 NVL burden and trends by country/territory

In 2021, South Asia reported the highest ASPR of NVL at 20,747.0 (95% UI: 15,692.2–26,642.3), contrasting with the Nordic Region, which had the lowest ASPR of 6,057.4 (95% UI: 4,486.6–8,160.3). For the age-standardized DALY rate, South Asia also ranked highest at 208.0 (95% UI: 94.1–378.4), while the Gulf Cooperation Council exhibited the lowest burden at 93.8 (95% UI: 42.0–181.9). Between 1990 and 2021, South Asia experienced the fastest rise in ASPR (EAPC: 2.37, 95% CI: 2.03–2.71), followed by the Commonwealth (EAPC = 2.00, 95% CI: 1.72–2.27). Similarly, the age-standardized DALY rate increased most sharply in South Asia (EAPC: 2.43, 95% CI: 2.08–2.78) and the Commonwealth (EAPC = 2.04, 95% CI: 1.76 to 2.32). In contrast, the European Union showed minimal changes in ASPR (EAPC: 0.00, 95% CI: −0.03 to 0.03), and the Gulf Cooperation Council experienced the largest decline in the age-standardized DALY rate (EAPC: −0.06, 95% CI: −0.07 to −0.05), reflecting divergent regional trajectories in disease burden management (Supplementary Table S2).

**Figure 3:**
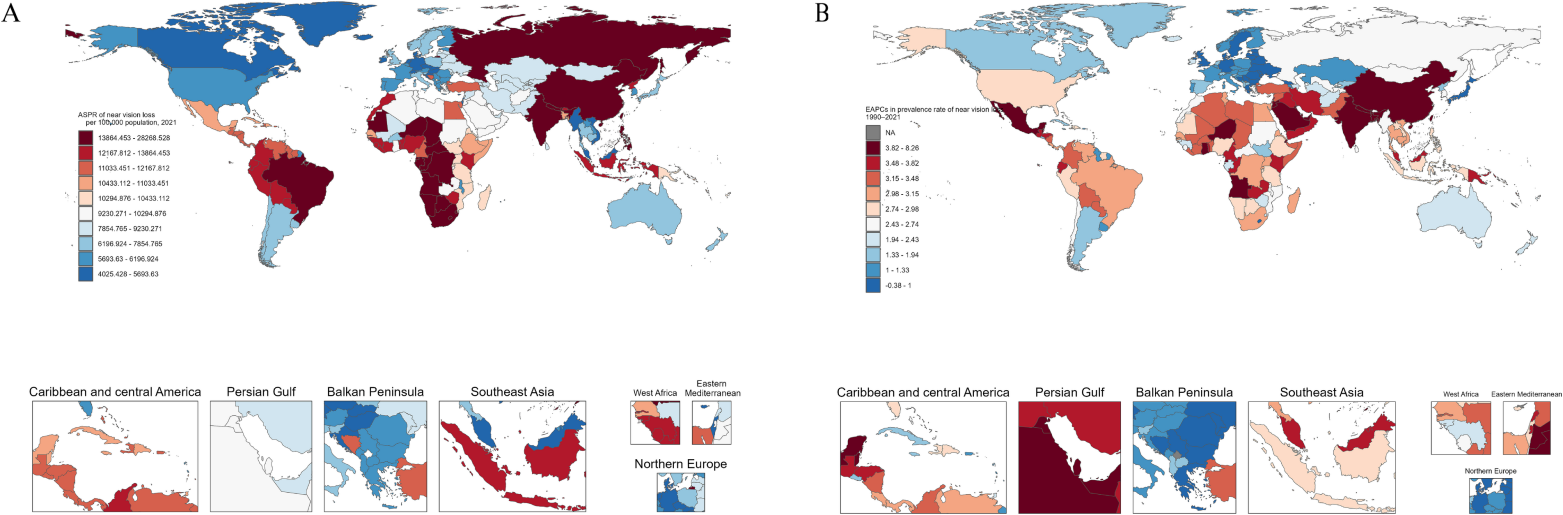
Global maps of NVL prevalence in 2021 **(A)** and the EAPC from 1990 to 2021 **(B)**. EAPC, estimated annual percentage change.

Among the 204 countries and territories, South Africa exhibited the highest ASPR in 2021 at 28,268.5 (95% UI: 22,241.3–35,472.4), whereas Malaysia recorded the lowest ASPR at 4,025.4 (95% UI: 2,982.8–5,390.4). For age-standardized DALYs, South Africa also had the highest rate at 283.9 (95% UI: 127.7 to 545.6), whereas Myanmar showed the lowest age-standardized DALYs at 49.8 (95% UI: 22.8–95.9). From 1990 to 2021, India demonstrated the most rapid increase in ASPR (EAPC: 2.68, 95% UI: 2.30–3.06), followed by China (EAPC: 1.77, 95% UI: 1.58–1.97), while Dominica experienced the largest decline (EAPC: −0.04, 95% CI: −0.085 to 0.002). Similarly, India displayed the steepest annualized rise in age-standardized DALYs (EAPC: 2.68, 95% CI: 2.30–3.06), contrasting with Germany, which had the most significant annualized reduction (EAPC: −0.08, 95% CI: −0.09 to −0.08) (Figures 3A,B and Supplementary Table S3).

### 3.3 NVL burden by SDI

**Figure 4:**
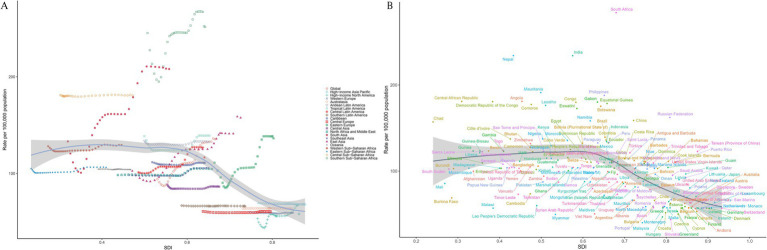
Age-standardized DALYs from 1990 to 2021 stratified by the SDI for 21 GBD regions **(A)** and 204 GBD countries and territories **(B)**. SDI, Socioeconomic Demographic Index; DALYs, disability-adjusted life years.

At the regional level, we observed an inverted L-shaped relationship between SDI and the prevalence of NVL and DALYs from 1990 to 2021. At the regional level, the SDI showed a significant negative correlation with NVL prevalence (*p* = −0.57, *p* < 0.0001) and DALYs (*p* = −0.56, *p* < 0.0001) (Figure 4A). At the national level, there was a significant negative association between SDI and prevalence (Spearman’s *ρ* = −0.54, *p* < 0.0001) and between SDI and DALYs (Spearman’s ρ = −0.58, *p* < 0.0001) (Figure 4B).

### 3.4 Cross-country health inequality analysis

**Figure 5:**
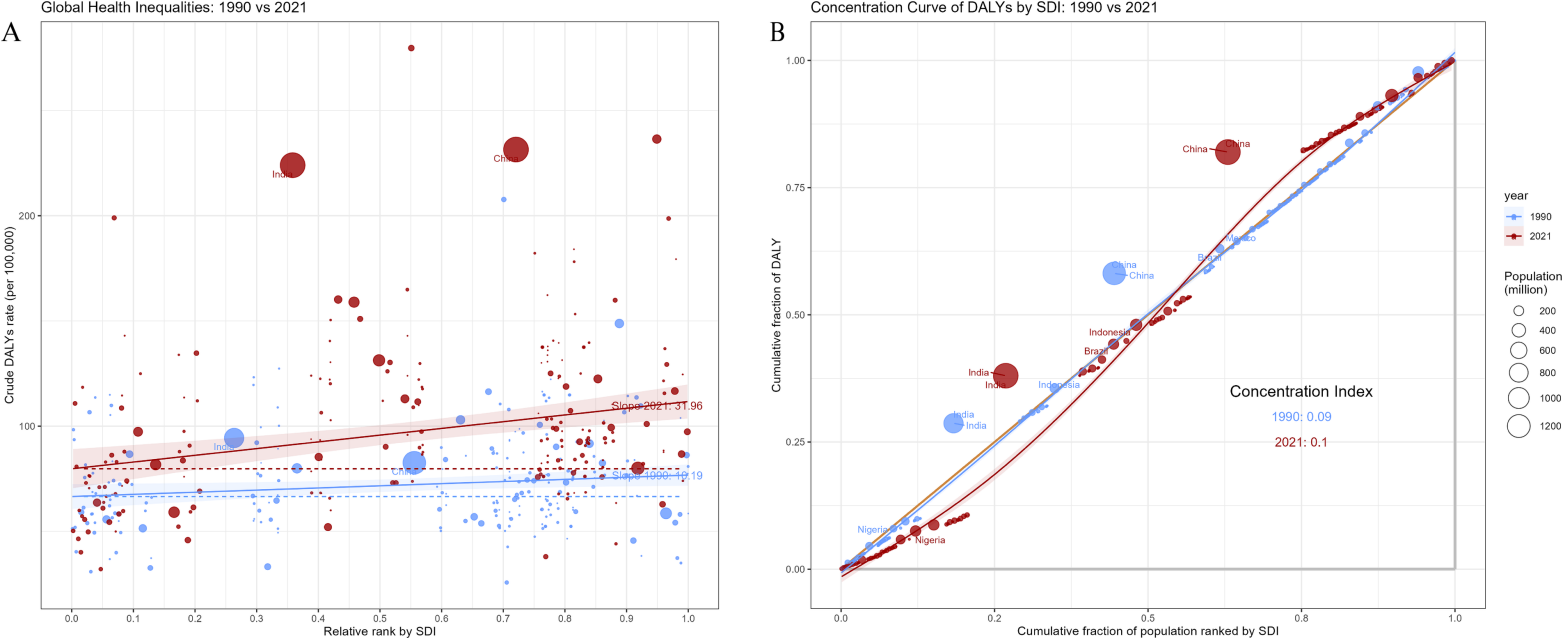
Regression curves **(A)** and concentration curves **(B)** of health inequality in DALYs of NVL; **(A)** illustrates the slope index of inequality, depicting the relationship between SDI and age-standardized DALYs rates for each condition, with points representing individual countries and territories sized by population; **(B)** presents the concentration index, which quantifies relative inequalities by integrating the area under the Lorenz curve, aligning DALYs distribution with population distribution by SDI. Blue represents data from 1990, and red represents data from 2021. DALYs, disability-adjusted life-years; SDI, socio-demographic index.

Regarding the NVL burden, we observed significant absolute inequalities associated with the SDI (Figure 5A). Countries and regions with a lower SDI disproportionately bore a higher burden (*p* < 0.0001). The magnitude of absolute inequality (SII, per 100,000) increased from 10.19 (95% CI: 0.11 to 20.47) in 1990 to 31.96 (95% CI: 17.83 to 48.39) in 2021. The concentration index for DALYs showed no significant changes in 2021 (Figure 5B and Supplementary Table S4).

### 3.5 Frontier analysis and decomposition analysis of the change in DALYs

**Figure 6:**
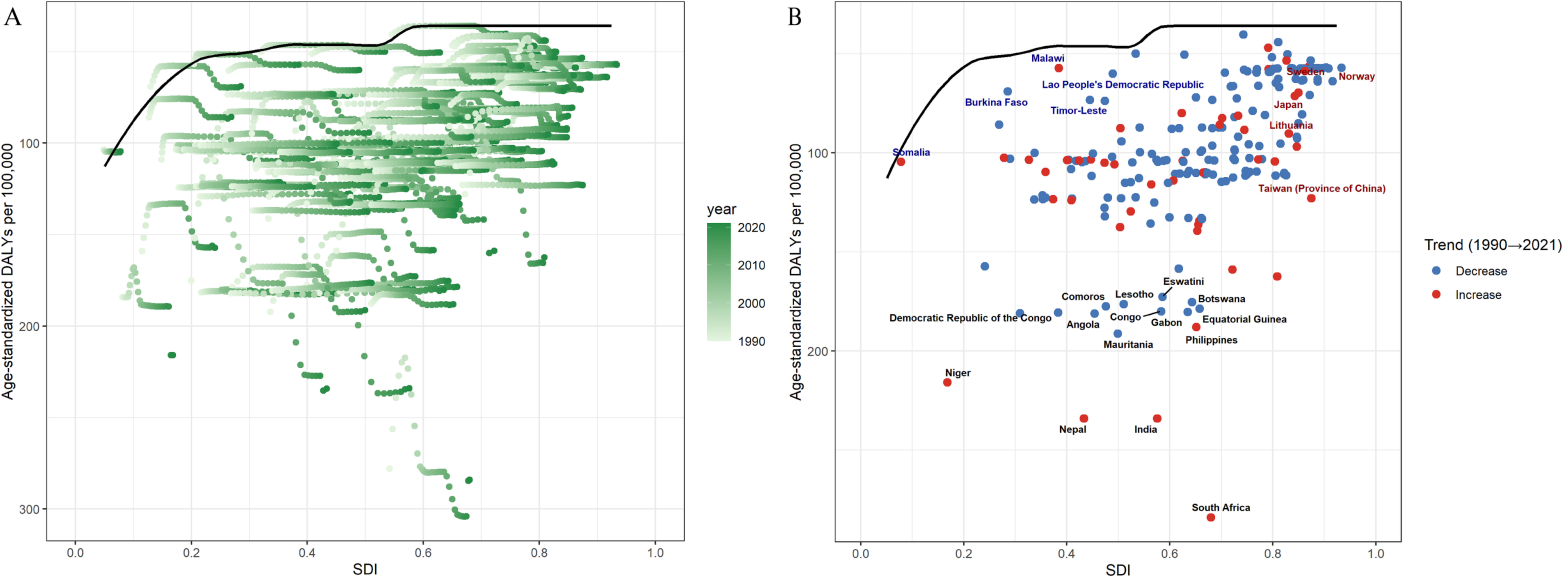
Frontier analysis of the relationship between SDI and DALYs for 204 countries and territories with respect to NVL. In **(A)**, the change from light green (1990) to dark green (2021) represents the shift over time. In **(B)**, each point represents a specific country or region in 2021, with borderlines shown in black. The 15 countries and territories with the greatest differences from the borderlines are marked in black. Blue represents countries and territories in low SDI with the smallest differences from the border, while red represents countries and territories in high SDI with the greatest differences. The direction of change in DALYs from 1990 to 2021 is indicated by the color of the points, with blue points representing a decrease and red points representing an increase.

Using data from 1990 to 2021, a frontier analysis based on DALYs and SDI was conducted to explore the potential for improvement in NVL, considering the development levels of the countries and territories (Figures 6A,B and Supplementary Table S5). The 15 countries and territories with the greatest potential for improvement are South Africa, India, Nepal, Niger, the Philippines, Mauritania, Gabon, the Congo, Equatorial Guinea, Botswana, Eswatini, Angola, the Democratic Republic of the Congo, Comoros, and Lesotho. Frontier countries and territories with a low SDI include Burkina Faso, Malawi, Mali, Myanmar, and Somalia. Considering their development levels, high-SDI countries and territories with relatively high improvement potential include Taiwan (a Province of China), Lithuania, Japan, Sweden, and Norway. Frontier analysis revealed the potential for improvement in reducing the NVL burden across countries and territories.

**Figure 7:**
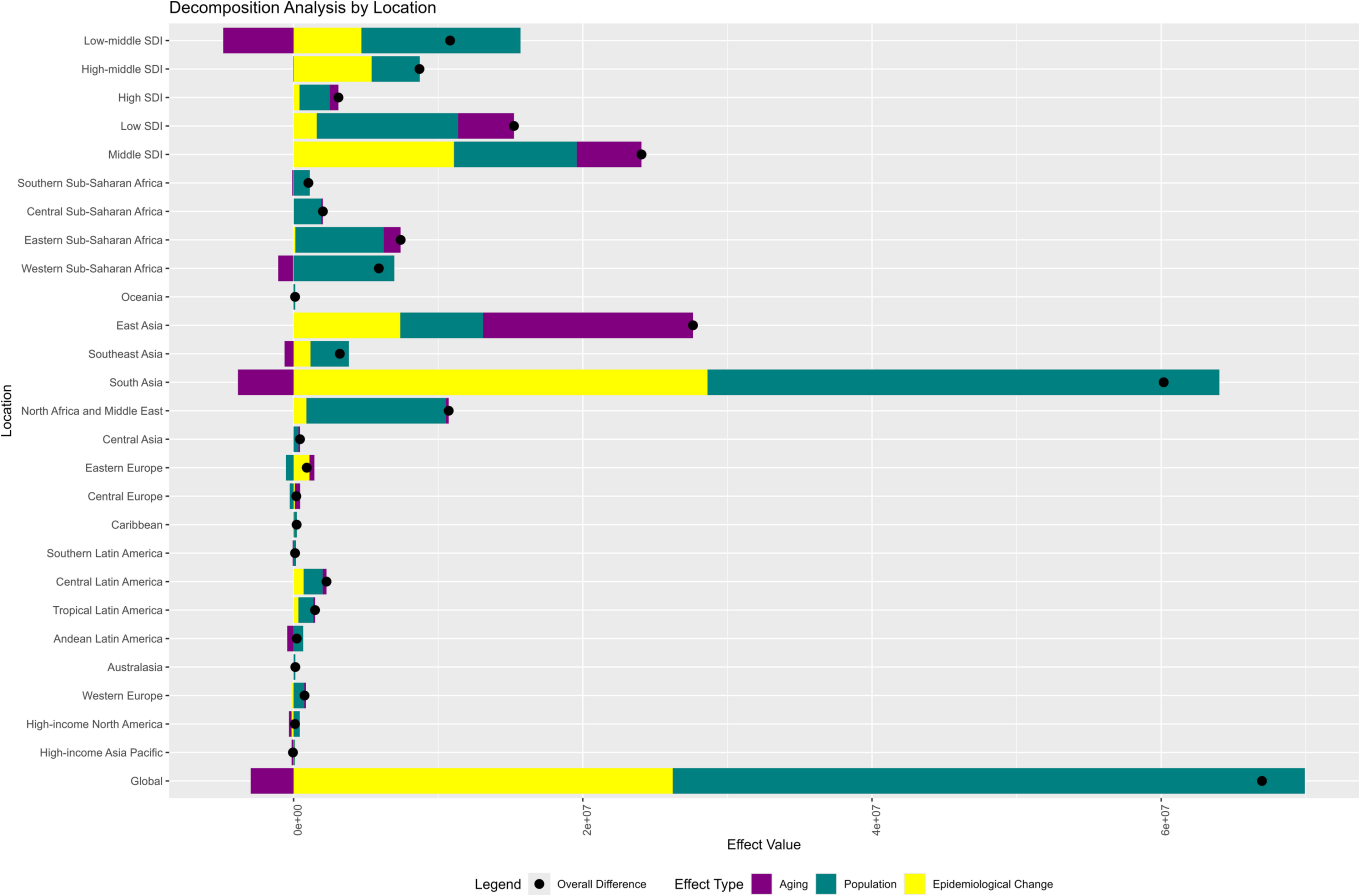
Decomposition of NVL DALY changes (1990–2021) by aging, population growth, and epidemiological transition across global regions. Black dots indicate total changes attributed to all three factors. SDI, Sociodemographic Index.

The decomposition analysis highlighted the distinct contributions of aging, population growth, and epidemiological changes to the NVL burden across regions. Globally, population growth (+65.27%) and epidemiological changes (+39.16%) drove the increase in DALYs, whereas aging marginally reduced the burden (−4.43%). South Asia exhibited the highest regional burden, dominated by population growth (+58.83%) and epidemiological changes (+47.57%), with aging offsetting a small fraction (−6.4%). Strikingly, the high-income Asia-Pacific region had an exceptionally high contribution from population aging (+303.17%), indicating that demographic aging has significantly increased the burden. In the SDI categories, Low-middle SDI regions experienced the largest population-driven increase (+101.7%), whereas High-middle SDI areas were predominantly influenced by epidemiological changes (+61.99%) (Figure 7 and Supplementary Table S6).

### 3.6 Future forecasts of global burden of NVL

**Figure 8:**
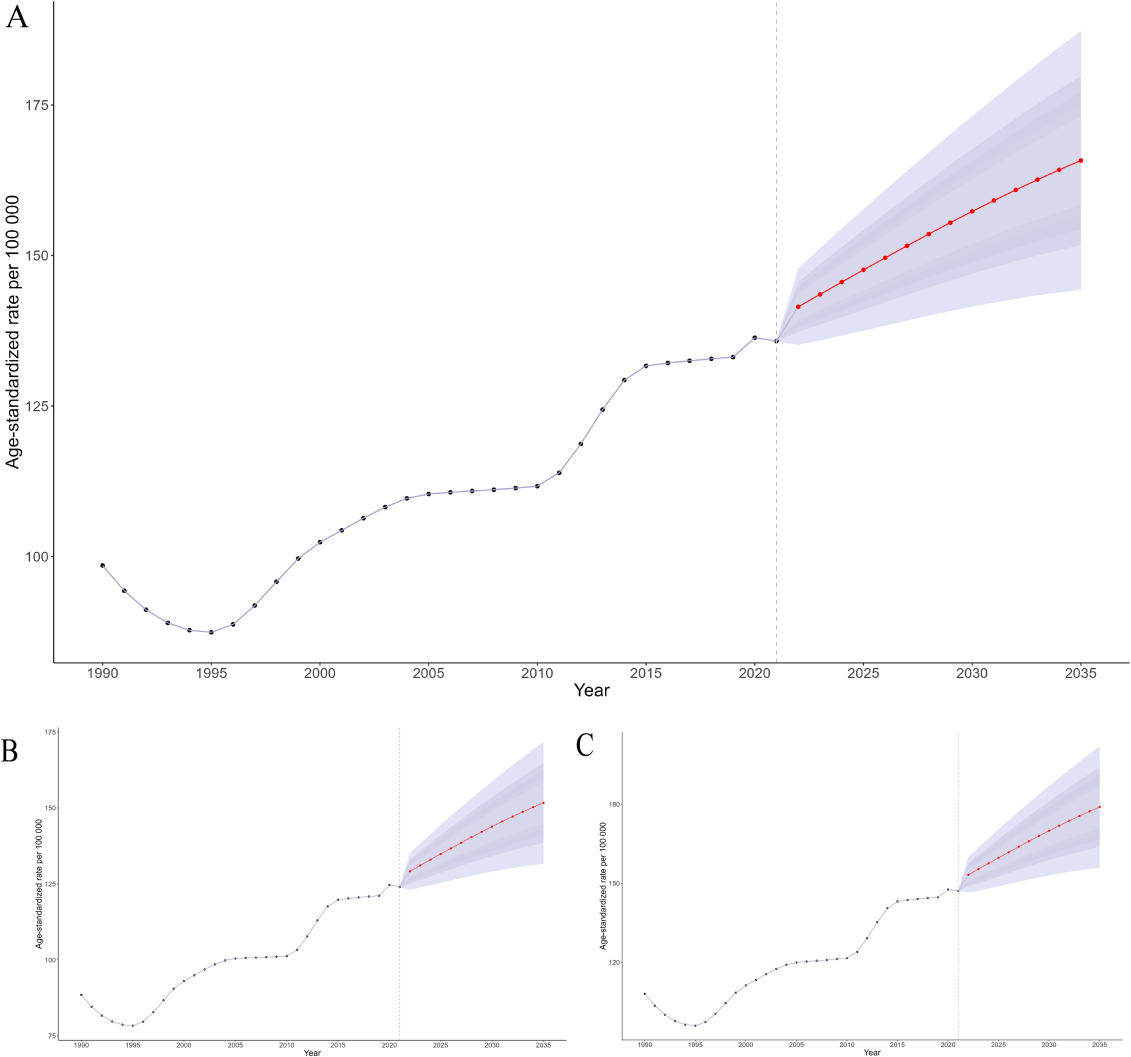
Future projections and temporal trends in ASPR from 1990 to 2035 for global **(A)**, male **(B)**, and female **(C)**.

The ASPR of NVL is projected to increase to 16,464.10 by 2035. Specifically, males are expected to demonstrate an ASPR elevation of 14,994.32, whereas females are predicted to experience a more pronounced surge, reaching 17,838.88. Concurrently, the age-standardized DALYs for NVL are anticipated to rise by 165.78 during the same period. Notably, across all evaluated metrics, the female cohorts consistently exhibit higher rates than their male counterparts (Figure 8 and Supplementary Table S7).

## 4 Discussion

This study conducted a systematic analysis of the GBD 2021 data on NVL and its epidemiological determinants, integrating the dimensions of socioeconomic development, population aging, and healthcare policies to inform evidence-based optimization of intervention strategies. The results demonstrated a substantial escalation in the global NVL burden, with the number of prevalent cases increasing dramatically from 428.0 million to 1.155 billion. Population growth accounted for 65.3% of the DALY variation, whereas the contribution of population aging was negligible (−4.4%). Furthermore, females exhibited a persistently higher ASPR than males. These findings suggest that the global burden of NVL is predominantly attributable to population growth and shows strong associations with sex disparities, socioeconomic development status, and regional healthcare policy frameworks.

Globally, NVL, predominantly uncorrected presbyopia, constitutes a major proportion of vision impairment. In 2019, DALYs from NVL accounted for 21.7% of the DALYs due to blindness and vision loss, approaching cataracts (29.6%), uncorrected distance refractive error (29.1%), far exceeding glaucoma (3.3%), and age-related macular degeneration (2.5%) (26). Uncorrected presbyopia imposes a population health burden comparable to that of major blinding diseases through limitations in reading, close work, and daily activities, underscoring the need to prioritize simple near-vision correction.

The analysis demonstrated significantly elevated prevalence rates and DALY burdens of NVL among females compared with males, aligning with established epidemiological patterns in ophthalmology (2, 27–30). This disparity is hypothesized to result from a confluence of biological determinants and structural health inequities. First, longer life expectancy leads to greater cumulative exposure to presbyopia, thereby resulting in a higher number of older women at risk of near-vision impairment (NVL) in the population (31). Second, unequal access to eye care between sexes is likely to play a role (32). In many low-resource settings, women face greater barriers to obtaining refractive correction due to lower incomes, less decision-making power, or cultural norms that prioritize men’s health needs (33–35). Third, cyclic estrogen variations such as those during menstruation (36), gestation (37), and menopause (38) have been mechanistically linked to refractive instability through corneal biomechanical alterations. Accordingly, gender-responsive delivery models that integrate refraction and on-site subsidized or free spectacle provision into women’s health services and community organizations can increase coverage, lower access costs, and prevent exclusion.

Notably, the epidemiological transition manifested as a 5-year advancement in the peak prevalence age, shifting from 55–59 years in 1990 to 50–54 years by 2021. One hypothesis is that prolonged near work (such as extensive use of smartphones, computers, and other digital devices) may be placing greater accommodative demands on the eyes from a younger age, leading to “accommodation fatigue” or earlier functional presbyopia (39). Modern lifestyles may accelerate the manifestation of presbyopic symptoms. Increased near work exposure could further amplify the global NVL burden, possibly affecting people in their 40s or late 30s with symptomatic near-vision strain.

This study elucidated the complex association between SDI and NVL disease burden. At the regional level, the SDI demonstrated an inverted L-shaped relationship with both NVL prevalence and DALYs, characterized by three distinct phases: a steep increase in disease burden at lower SDI values, decelerated growth at moderate SDI levels, and eventual stabilization in high-SDI regions. These patterns correlate with global disparities in refractive care, where over 90% of individuals in rural areas of low- and middle-income countries lack corrective interventions compared with about 40% in urban or high-income settings (19). It is important to note that this socioeconomic gradient manifests not only in healthcare resource allocation but may also influence disease progression through multiple risk exposure pathways. A systematic association exists between lower socioeconomic status and factors such as uncorrected refractive errors, nutritional deficiencies, elevated ultraviolet radiation exposure, air pollution exposure, and higher prevalence of systemic comorbidities (40–42). The synergistic interactions between these factors likely exacerbate geographic disparities in the prevalence of NVL. Our analysis identifies SDI > 0.6 as a critical threshold for achieving substantial NVL burden reduction through basic health care coverage.

Notably, absolute inequalities in the NVL burden remain stark: the magnitude of absolute inequality between the highest and lowest SDI regions has widened from 10.19 (1990) to 31.96 (2021), driven by population growth (65.3% contribution) and delayed corrective interventions in low-SDI settings. Longitudinal data show that although low- to middle-SDI countries and territories have made significant progress in reducing blindness over the last three decades, persistent health inequalities remain evident (43). The factors contributing to this disparity are multifaceted. Rapid population growth and aging in low-SDI regions have greatly increased the number of NVL cases. Second, the persistent underprovision of vision correction services within these regions compounded this issue. Countries and territories with high SDI have been more effective in addressing presbyopia (through better health care access and higher eyeglass uptake). Access to near-vision correction in low-SDI settings is constrained by interrelated economic, cultural, and geographic barriers. Economically, even low-cost spectacles are unaffordable where health spending is predominantly out of pocket and subsidy mechanisms are absent. For instance, in rural India, cost is the most commonly cited reason for not obtaining glasses despite documented productivity gains from near-vision correction (44). Individuals earning less than $53.0 per month versus more than $107.1 per month had 48-fold higher odds of purchasing spectacles, an income gradient that likely contributed to the high burden of uncorrected presbyopia in low- and middle-income countries and territories (45). Financial constraints are compounded by a tendency to prioritize other household needs over ocular health. Culturally, low awareness and limited health literacy foster perceptions that presbyopia is an inevitable consequence of aging rather than a treatable impairment, and the stigma surrounding spectacle wear (e.g., beliefs that glasses weaken the eyes or denote frailty) deters uptake, as reported in Nepal (10). Geographic disparities further impede access because eye care services and optical shops are concentrated in urban centers, leaving rural populations underserved (46). Overall, these barriers—financial, informational, and service availability—help explain why presbyopia remains substantially undercorrected in low-SDI regions.

Based on data from 1990 to 2021, a frontier analysis using DALYs and SDI highlighted the significant potential for reducing the burden of NVL in different countries and territories. South Africa, India, and 15 other countries and territories had the largest gaps in improvement potential, reflecting the uneven distribution of the global disease burden, with low- and middle-income areas struggling because of limited resources. Interestingly, some high-SDI regions also showed a high improvement potential, indicating the need for an in-depth exploration of health system vulnerabilities in middle- and high-SDI countries and territories. Notably, even within these high-income settings, direct costs—including expenses related to accessing eye care services, transportation for appointments, and pharmaceutical interventions—remain critical barriers to equitable visual health, particularly for populations in rural areas or with lower socioeconomic status (47). Targeted policies to address disparities in access to vision care in high-SDI countries and territories are required. Priority actions include expanding insurance coverage for refractive services, deploying mobile clinics in underserved rural areas, and subsidizing transport for low-income patients to secure equitable access for marginalized groups.

Decomposition analysis revealed heterogeneous drivers underlying the evolving disease burden of NVL. Globally, population growth accounted for 65.3% of the DALY changes, far exceeding the contributions of aging (−4.4%) and epidemiological shifts (39.2%). This predominance is closely tied to the cumulative burden of uncorrected cases in low-SDI regions owing to insufficient coverage of refractive services. In sub-Saharan Africa, the population-driven effect exceeds 100%, whereas epidemiological changes exhibit a slightly negative impact. Notably, studies indicate that the accelerated development of optometric education in sub-Saharan Africa has enhanced the availability of a skilled workforce for delivering quality eye care, thereby improving access to refractive services (48). These findings underscore that while progress in optometric education has partially alleviated service gaps in the region, there is an urgent need to synchronize primary eye care network development with persistently high population growth rates to address the long-term imbalance between demographic expansion and resource provision. The BAPC projections indicated a steepening trajectory of the NVL burden. This escalation reflects multiple factors: population growth in low-SDI regions, accelerated aging in middle-income regions, and persistent under-correction of refractive errors. Low-SDI regions face dual pressures of population expansion and lagging service coverage.

A coordinated package—comprising mass provision of low-cost spectacles, community outreach, integration into primary care, and telemedicine-enabled screening—can deliver rapid and scalable gains in closing the near-vision care gap. A priority policy is the large-scale provision of low-cost spectacles. Given the minimal unit costs for ready-made readers, governments and nongovernmental organizations can efficiently deliver them to high-need communities through subsidies or free distribution. Even modest investments are likely to yield substantial returns by restoring near vision for work, learning, and daily functioning, thereby improving productivity and quality of life (23). Community-based programs are critical delivery channels for near vision care. Regular screening camps in rural settings and the incorporation of near-vision checks into existing outreach enable the rapid identification of presbyopia and same-day dispensing of spectacles. These models reduce geographic access barriers and reach populations that are otherwise missed by providing services directly to underserved communities. Integrating presbyopia screening and treatment into primary care is essential for the sustainability and equitable reach of the program. Consistent with the World Health Organization’s framework of integrated, people-centered eye care (IPEC), routine primary care visits should include a simple near-vision test and on-site dispensing of appropriate ready-made spectacles with referral for complex refractive needs. Embedding these steps into routine encounters identifies individuals who would not otherwise seek eye care because of their low awareness or competing priorities (32). Telemedicine- and smartphone-based examinations can narrow the refractive care gap, particularly in primary care and community settings. Low-cost applications and peripherals enable remote vision screening and basic ocular assessment with minimal infrastructure (49). Currently, smartphone-based mobile refractors are available. Luo et al. (50) validated a smartphone application for estimating myopic refractive error in 113 participants with ≤1.75 D astigmatism, demonstrating strong agreement with clinical refraction and autorefractor measurements and good test–retest reliability. For presbyopia, self-administered near-vision and simplified refraction tests can triage users into ready-made spectacles, with virtual optometrist follow-ups when necessary. When integrated into routine workflows, these tools achieve screening-level accuracy, extend reach to underserved areas, and support task sharing.

Although this study provided critical insights into the global epidemiology of NVL, it had three principal limitations. First, global NVL estimates are limited by cross-country differences in assessment and reporting, and widespread underdiagnosis, which together may lead to variability and underestimation. For example, the definition of “near vision loss” might not be perfectly uniform—some studies define it by the inability to read standard near print at 40 cm, while others define it by a certain dioptric requirement. We assumed comparability based on GBD standardized adjustments; however, residual differences may remain. Second, NVL is often underdiagnosed in communities that lack regular eye examinations. Many individuals do not realize that their vision can be corrected, and thus never report or seek care for near-vision difficulties. This underdiagnosis means that our NVL burden estimates might be conservative in some low-access settings because undetected cases would not have been counted in official data. Third, the temporal scope precluded a comprehensive assessment of the COVID-19 pandemic’s post-2020 impact, which has likely exacerbated healthcare disparities and disrupted refractive care continuity among vulnerable populations. Furthermore, the correlations observed in previous studies, such as the relationship between SDI and NVL prevalence, may not necessarily imply causality. In addition to these empirical and scope-related constraints, we note a further conceptual caveat related to our reliance on DALYs. Although DALYs remain a widely used and informative metric for cross-population comparison, disability scholars and public health ethicists have cautioned that DALY frameworks can inadvertently encode ableist assumptions by equating disability with diminished quality or value of life (51, 52). Accordingly, DALY-based estimates may understate the worth of years lived with disability and risk skewing priority-setting toward states deemed “healthier” by construction (53). Because our analysis relies on DALYs, our interpretations inherit this normative limitation. We believe this addition enhances the ethical transparency and conceptual balance of our study.

Looking ahead, reducing NVL will depend on implementation research that evaluates scalable service delivery packages across SDI strata, such as the mass provision of low-cost spectacles, community outreach, primary care integration, and teleophthalmology, while concurrently strengthening supply chain resilience and eye health workforce capacity. Priorities include standardizing near-vision metrics and surveillance systems, validating AI-enabled screening in real-world settings, and embedding explicit equity targets in financing mechanisms (e.g., insurance expansion and transport subsidies). Linking cost-effectiveness evidence to DALY reduction can reveal the benefits of package design. Co-designed approaches involving women and rural communities are essential for closing persistent access gaps.

## 5 Conclusion

Our analysis indicates that the global burden of NVL is driven primarily by population growth, with low SDI regions disproportionately affected because uncorrected cases accumulate under inadequate refractive service coverage. Women exhibited significantly higher ASPR than men, highlighting the disparities in sex-specific risk profiles and in societal resource allocation. These findings support a tiered response in which priority should be given to expanding essential refractive services and implementing gender-responsive policies (e.g., community-based vision screening for women) to reduce inequities and slow the emerging trend toward an earlier onset. Correcting presbyopia can yield substantial economic and social benefits through productivity and quality-of-life gains, underscoring the urgency of expanding access to near-vision services.

## Data Availability

The original contributions presented in the study are included in the article/Supplementary material, further inquiries can be directed to the corresponding authors.

## Footnotes

^1^ http://ghdx.healthdata.org/gbd-results-tool

## Glossary

NVL: near vision loss
GBD: Global Burden of Disease
DALYs: disability-adjusted life years
SDI: sociodemographic index
BAPC: Bayesian age–period–cohort
ASPR: age-standardized prevalence rate
UI: uncertainty interval
APC: annual percentage change
AAPC: average annual percentage change
EAPC: estimated annual percentage change
CI: confidence interval
GSM: grid search method
MSE: mean squared error
SII: slope index of inequality
RLM: robust linear regression model
LM: linear regression model
LOESS: locally weighted regression
RW2: second-order random walk
INLA: Integrated Nested Laplace Approximation
IHME: Institute for Health Metrics and Evaluation
