# Supplementary material for: Female predominance and socio-demographic inequalities in global near vision loss burden: projected trends and disparities from 1990 to 2035
Source: Front Public Health. 2025 Nov 3;13:1611433. doi: 10.3389/fpubh.2025.1611433 (PMC12620233; doi:10.3389/fpubh.2025.1611433)
Supplement: Supplementary file 2 [file Supplementary_file_1.docx]

**1. NVL_Supplement_TableBuilder.R**

library(data.table)

library(dplyr)

library(tidyr)

library(broom)

df <- fread("./data/raw_204_countries_unprocessed.csv")

df_pc <- fread("./data/pc.csv")

keys <- c("location_name","age_name","cause_name","metric_name")

df_num <- df %>%

filter(year %in% c(1990, 2021), sex_name == "Both") %>%

filter((age_name == "All ages" & metric_name == "Number") | (age_name != "All ages" & metric_name == "Rate")) %>%

mutate(num = sprintf("%.3f(%.3f-%.3f)", val, lower, upper)) %>%

select(all_of(keys), year, num) %>%

distinct() %>%

pivot_wider(names_from = year, values_from = num)

df_pc_tidy <- df_pc %>%

filter(measure_name == "DALYs (Disability-Adjusted Life Years)",

sex_name == "Both",

metric_name != "Percent") %>%

mutate(PC = sprintf("%.3f(%.3f-%.3f)", val, lower, upper)) %>%

select(all_of(keys), PC) %>%

distinct()

decx <- 2

df_eapc <- df %>%

filter(measure_name == "DALYs (Disability-Adjusted Life Years)",

sex_name == "Both",

metric_name != "Percent") %>%

group_by(across(all_of(keys))) %>%

do({

fit <- lm(log(val) ~ year, data = .)

ti <- tidy(fit, conf.int = TRUE)

row <- ti[ti$term == "year", c("estimate","conf.low","conf.high")]

eapc <- 100 * (exp(row$estimate) - 1)

lo <- 100 * (exp(row$conf.low) - 1)

hi <- 100 * (exp(row$conf.high) - 1)

tibble(EAPCs = sprintf(paste0("%.", decx, "f(%.", decx, "f,%.", decx, "f)"), eapc, lo, hi))

}) %>%

ungroup()

dfall <- df_num %>%

left_join(df_pc_tidy, by = keys) %>%

left_join(df_eapc, by = keys)

fwrite(dfall, "table1_204_countries.csv")

**2.NVL_Joinpoint_DALYs_Fig.R**

library(ggplot2)

library(dplyr)

library(scales)

library(ggrepel)

library(purrr)

apc <- read.csv("DALYs_APC.csv") %>%

mutate(

APC.Label = ifelse(APC.Significant == 1, paste0(round(val, 2), "*"), paste0(round(val, 2))),

sex_name = factor(sex_name, levels = c("Both", "Female", "Male")),

mid_year = (Segment.Start + Segment.End) / 2

)

prevalence <- read.csv("Global_DALYs (Disability-Adjusted Life Years)_joinpoint.csv") %>%

mutate(sex_name = factor(sex_name, levels = c("Both", "Female", "Male")))

apc <- apc %>%

mutate(

y_start = pmap_dbl(

list(sex_name, Segment.Start),

~ approx(

x = prevalence$year[prevalence$sex_name == ..1],

y = prevalence$val[prevalence$sex_name == ..1],

xout = ..2,

rule = 2

)$y

),

y_end = pmap_dbl(

list(sex_name, Segment.End),

~ approx(

x = prevalence$year[prevalence$sex_name == ..1],

y = prevalence$val[prevalence$sex_name == ..1],

xout = ..2,

rule = 2

)$y

),

base_y = pmap_dbl(

list(sex_name, mid_year),

~ approx(

x = prevalence$year[prevalence$sex_name == ..1],

y = prevalence$val[prevalence$sex_name == ..1],

xout = ..2,

rule = 2

)$y

),

nudge_y = map_dbl(

sex_name,

~ 0.03 * diff(range(prevalence$val[prevalence$sex_name == .x]))

)

)

sex_colors <- c("Both" = "#1f77b4", "Female" = "#ff7f0e", "Male" = "#2ca02c")

p <- ggplot() +

geom_line(

data = prevalence,

aes(x = year, y = val, color = sex_name),

linewidth = 0.8

) +

geom_segment(

data = apc,

aes(x = Segment.Start, xend = Segment.End, y = y_start, yend = y_end, color = sex_name),

linewidth = 1.2,

linetype = "11",

alpha = 0.7

) +

geom_vline(

data = apc %>% filter(Segment.Start > 1990),

aes(xintercept = Segment.Start),

color = "grey50",

linetype = "dotted",

linewidth = 0.6

) +

geom_text_repel(

data = apc,

aes(x = mid_year, y = base_y, label = APC.Label, color = sex_name),

size = 3.2,

direction = "y",

nudge_y = apc$nudge_y,

segment.size = 0.2,

show.legend = FALSE

) +

scale_color_manual(

name = "Sex",

values = sex_colors,

labels = c("Both sexes", "Female", "Male")

) +

scale_x_continuous(

breaks = seq(1990, 2020, 5),

limits = c(1990, 2021),

expand = expansion(0.01)

) +

scale_y_continuous(

name = "Age-standardized DALYs rate per 100,000",

labels = number_format(accuracy = 1),

expand = expansion(mult = c(0.05, 0.1))

) +

labs(x = "Year") +

theme_classic(base_size = 12) +

theme(

panel.grid.major.y = element_line(color = "grey90", linewidth = 0.3),

legend.position = c(0.95, 0.15),

legend.justification = c(1, 0),

legend.background = element_rect(fill = alpha("white", 0.8)),

plot.title = element_blank()

)

ggsave("Fig1_DALYs_Joinpoint.tiff", plot = p, width = 24, height = 16, units = "cm", dpi = 600)

**3.NVL_DALY_WorldMap_Insets.R**

library(data.table)

library(dplyr)

library(ggplot2)

library(sf)

library(patchwork)

df <- fread("./data/nvl_merged.csv")

load("./data/GBD.Rdata") # expects df_world (sf) and namex (location_id, location)

labelx <- "DALYs (Disability-Adjusted Life Years)"

dfx <- df %>%

filter(

year == 2021,

measure_name == labelx,

sex_name == "Both",

age_id == 27,

metric_name == "Rate",

cause_name == "Near vision loss"

)

df_world <- df_world %>%

mutate(location_id = if_else(name_long == "India", 163L, location_id))

dfplot <- dfx %>%

filter(location_id %in% namex$location_id) %>%

select(location_id, val) %>%

left_join(namex, by = "location_id")

ASR <- dfplot %>% select(location_id, location, val)

df_asr <- left_join(df_world, ASR, by = "location_id")

br <- quantile(ASR$val, probs = seq(0, 1, length.out = 11), na.rm = TRUE)

br <- unique(br)

if (length(br) < 2) {

r <- range(ASR$val, na.rm = TRUE)

br <- seq(r[1], r[2], length.out = 11)

}

lab <- sprintf("%.3f - %.3f", head(br, -1), tail(br, -1))

df_asr <- df_asr %>%

mutate(asr_cut = cut(val, breaks = br, include.lowest = TRUE, labels = lab)) %>%

filter(!is.na(asr_cut))

n_bins <- length(levels(df_asr$asr_cut))

pal <- colorRampPalette(c("#67001f","#b2182b","#d6604d","#f4a582","#fddbc7",

"#f7f7f7","#d1e5f0","#92c5de","#4393c3","#2166ac"))(n_bins)

p_main <- ggplot(df_asr) +

geom_sf(aes(geometry = geometry, fill = asr_cut), linewidth = 0.1) +

scale_fill_manual(values = rev(pal), guide = guide_legend(reverse = TRUE),

name = "Age-standardized DALY rate per 100,000 (2021)") +

theme_void(base_size = 11) +

theme(legend.position = c(0.13, 0.29),

legend.background = element_blank(),

legend.key = element_blank(),

legend.title = element_text(size = 8),

legend.text = element_text(size = 8))

theme_map_sub <- theme_void() +

theme(text = element_text(size = 12),

legend.position = "none",

plot.background = element_rect(fill = "transparent"),

plot.title = element_text(vjust = 0.01, hjust = 0.5, size = 10))

sub1 <- p_main + coord_sf(xlim = c(-92, -59), ylim = c(7, 28), expand = FALSE) + ggtitle("Caribbean and Central America") + theme_map_sub

sub2 <- p_main + coord_sf(xlim = c(45, 55.8), ylim = c(21, 31.5), expand = FALSE) + ggtitle("Persian Gulf") + theme_map_sub

sub3 <- p_main + coord_sf(xlim = c(12.5, 32), ylim = c(34.5, 50), expand = FALSE) + ggtitle("Balkan Peninsula") + theme_map_sub

sub4 <- p_main + coord_sf(xlim = c(94.9, 119.1),ylim = c(-9.2, 9), expand = FALSE) + ggtitle("Southeast Asia") + theme_map_sub

sub5 <- p_main + coord_sf(xlim = c(-17.8, -7), ylim = c(6.5, 15.8), expand = FALSE) + ggtitle("West Africa") + theme_map_sub

sub6 <- p_main + coord_sf(xlim = c(30.5, 38.5), ylim = c(28.4, 35.9), expand = FALSE) + ggtitle("Eastern Mediterranean") + theme_map_sub

sub7 <- p_main + coord_sf(xlim = c(4.7, 27.5), ylim = c(48, 59), expand = FALSE) + ggtitle("Northern Europe") + theme_map_sub

plot_top <- (sub1 + sub2 + sub3 + sub4) + plot_layout(nrow = 1)

plot_bottom <- (sub5 | sub6) / sub7 + plot_layout(heights = c(2.8, 2.5))

insets <- plot_top | plot_bottom + plot_layout(widths = c(1, 15))

px <- p_main / insets + plot_layout(heights = c(2, 1), widths = c(2, 1.2))

ggsave("NVL_DALY_map.pdf", px, width = 12, height = 10)

fwrite(st_drop_geometry(df_asr), "NVL_DALY_df_asr.csv")

fwrite(dfplot, "NVL_DALY_dfplot.csv")

4. **NVL_DALY_SDI_Scatter.R**

library(data.table)

library(dplyr)

library(ggplot2)

library(ggrepel)

rm(list = ls())

load("./data/GBD.Rdata") # must contain SDI2019 (location_id, location_name, sdi)

df <- fread("./data/DALY_NVL_204.csv")

label_dalys <- "DALYs (Disability-Adjusted Life Years)"

dfx <- df %>%

filter(

measure_name == label_dalys,

sex_name == "Both",

cause_name == "Near vision loss",

metric_name == "Rate",

year == 2021

) %>%

select(location_id, location_name, val, year)

SDI_joined <- SDI2019 %>%

left_join(dfx, by = "location_id") %>%

filter(!is.na(val), !is.na(sdi))

sp <- cor.test(SDI_joined$sdi, SDI_joined$val, method = "spearman")

r <- as.numeric(sp$estimate)

p <- as.numeric(sp$p.value)

subtitle_str <- sprintf("r=%.4f, p=%.3e", r, p)

p1 <- ggplot(SDI_joined, aes(x = sdi, y = val)) +

geom_point(aes(color = location_name), size = 0.8) +

geom_smooth(method = "loess", se = TRUE, color = "#708090") +

geom_text_repel(aes(label = location_name, color = location_name),

size = 3.0, max.overlaps = 60, segment.size = 0.2, show.legend = FALSE) +

labs(

x = "SDI (2019)",

y = "DALY rate per 100,000",

subtitle = subtitle_str

) +

theme_bw() +

theme(

legend.position = "none",

panel.border = element_blank(),

panel.grid.major = element_blank(),

panel.grid.minor = element_blank(),

axis.line = element_line(colour = "black")

) +

scale_x_continuous(limits = c(0.2, 1.0), breaks = seq(0.2, 1.0, 0.1))

ggsave("NVL_DALY_SDI_scatter.pdf", plot = p1, width = 16, height = 10)

write.table(

data.frame(Measure_Name = "Near vision loss (DALY rate, 2021)", Spearman_R = r, p_value = p),

file = "NVL_DALY_SDI_correlation.txt",

row.names = FALSE, col.names = TRUE, sep = "\t", quote = FALSE

)

ggsave(

filename = "NVL_DALY_SDI_scatter.tiff",

plot = p1,

device = "tiff",

dpi = 300,

width = 12,

height = 8,

units = "in",

compression = "lzw"

)

**5.NVL_AgeSex_Prevalence_NumberRate_Overlay.R**

library(data.table)

library(dplyr)

library(ggplot2)

library(scales)

df <- fread("./data/nvl_merged.csv")

dfx <- df %>%

filter(

measure_name == "Prevalence",

location_id %in% 1,

year == 2021,

sex_name %in% c("Female", "Male"),

cause_name == "Near vision loss",

metric_name %in% c("Number", "Rate"),

age_id %in% c(1, 5:20, 30, 31, 32, 235)

) %>%

mutate(

age_name = if_else(age_name %in% c("80-84", "85-89", "90-94"),

paste(age_name, "years"), age_name),

sex_name = factor(sex_name, levels = c("Female", "Male"))

)

age_levels <- dfx %>%

distinct(age_id, age_name) %>%

arrange(age_id) %>%

pull(age_name)

dfx <- dfx %>% mutate(age_name = factor(age_name, levels = age_levels))

df_number <- dfx %>% filter(metric_name == "Number")

df_rate <- dfx %>% filter(metric_name == "Rate")

max_number <- max(df_number$val, na.rm = TRUE)

max_rate <- max(df_rate$val, na.rm = TRUE)

scaling_factor <- max_number / max_rate

if (!is.finite(scaling_factor) || scaling_factor <= 0) scaling_factor <- 1

pd <- position_dodge(width = 0.9)

cols <- c("Female" = "#D77A7A", "Male" = "#6C8EBF")

p <- ggplot() +

geom_col(

data = df_number,

aes(x = age_name, y = val, fill = sex_name),

position = pd, width = 0.8

) +

geom_errorbar(

data = df_number,

aes(x = age_name, ymin = lower, ymax = upper, group = sex_name),

position = pd, width = 0.25

) +

geom_line(

data = df_rate,

aes(x = age_name, y = val * scaling_factor, color = sex_name, group = sex_name),

linewidth = 0.9, position = pd

) +

geom_ribbon(

data = df_rate,

aes(x = age_name,

ymin = lower * scaling_factor,

ymax = upper * scaling_factor,

fill = sex_name,

group = sex_name),

alpha = 0.15, position = pd

) +

scale_fill_manual(values = cols, name = "Sex") +

scale_color_manual(values = cols, name = "Sex") +

scale_y_continuous(

name = "Number (K)",

labels = label_number(scale = 1e-3, suffix = "K"),

sec.axis = sec_axis(~ . / scaling_factor, name = "Rate per 100,000")

) +

labs(x = "Age group") +

theme_classic(base_size = 12) +

theme(

axis.title.x = element_text(size = 14),

axis.title.y = element_text(size = 14),

axis.text.x = element_text(size = 10.6, angle = 65, hjust = 1),

axis.text.y = element_text(size = 10.6),

legend.title = element_text(size = 14),

legend.text = element_text(size = 10.6)

)

ggsave(

filename = "Age_Prevalence_NumberRate_Overlay.pdf",

plot = p, width = 12, height = 8, units = "in", bg = "transparent"

)

ggsave(

filename = "Age_Prevalence_NumberRate_Overlay.tiff",

plot = p, width = 12, height = 8, units = "in",

dpi = 300, device = "tiff", compression = "lzw", bg = "transparent"

)

fwrite(df_number, "Age_Prevalence_Number_Data.csv")

fwrite(df_rate, "Age_Prevalence_Rate_Data.csv")

**6. NVL_Inequality_SDI_Concentration_1990_2021.R**

# Libraries

library(data.table)

library(dplyr)

library(tidyr)

library(ggplot2)

library(ggrepel)

library(MASS)

library(splines)

library(mgcv)

library(broom)

library(scales)

# ----------------------------- Inputs -----------------------------

# Provide these files/folders:

# ./data/country_burden.csv (GBD outputs by country)

# ./data/SDI.csv (wide: columns 1990:2021)

# ./data/GBD_population/ (folder with population CSVs)

burden <- fread("./data/country_burden.csv")

sdi_w <- read.csv("./data/SDI.csv", check.names = FALSE)

pop_dir <- "./data/GBD_population"

# ----------------------------- SDI long ---------------------------

sdi <- sdi_w %>%

pivot_longer(cols = `1990`:`2021`, names_to = "year", values_to = "sdi") %>%

rename(location_name = 1) %>%

mutate(year = as.integer(year))

selected_countries <- unique(sdi$location_name)

# ----------------------------- Burden filter ----------------------

burden2 <- burden %>%

filter(

location_name %in% selected_countries,

sex_name == "Both",

age_name == "All ages",

cause_name == "Near vision loss",

measure_name == "DALYs (Disability-Adjusted Life Years)",

metric_name %in% c("Rate", "Number"),

year >= 1990, year <= 2021

)

# ----------------------------- Population ------------------------

pop_files <- list.files(pop_dir, full.names = TRUE)

population <- rbindlist(lapply(pop_files, fread), use.names = TRUE, fill = TRUE)

pop_all <- population %>%

select(location_name, sex_name, age_name, year, val) %>%

filter(age_name == "All ages") %>%

select(location_name, sex_name, year, pop = val)

if (!"Both" %in% unique(pop_all$sex_name)) {

pop_all <- pop_all %>%

group_by(location_name, year) %>%

summarise(pop = sum(pop, na.rm = TRUE), .groups = "drop") %>%

mutate(sex_name = "Both")

} else {

pop_all <- pop_all %>%

filter(sex_name == "Both")

}

# ----------------------------- Merge core data --------------------

dat <- burden2 %>%

left_join(sdi, by = c("location_name", "year")) %>%

left_join(pop_all %>% select(location_name, year, pop),

by = c("location_name", "year")) %>%

filter(!is.na(val), !is.na(pop), !is.na(sdi))

global_pop <- dat %>%

group_by(year) %>%

summarise(pop_global = sum(pop, na.rm = TRUE), .groups = "drop")

dat <- dat %>% left_join(global_pop, by = "year")

# ----------------------------- Weighted rank by SDI ---------------

ranked <- dat %>%

arrange(year, sdi) %>%

group_by(year) %>%

mutate(cum_pop = cumsum(pop),

weighted_order = (cum_pop - pop/2) / pop_global) %>%

ungroup()

# ----------------------------- Robust regressions (1990/2021) ----

rate_1990 <- ranked %>% filter(metric_name == "Rate", year == 1990)

rate_2021 <- ranked %>% filter(metric_name == "Rate", year == 2021)

fit_rlm_1990 <- if (nrow(rate_1990) > 0) rlm(val ~ weighted_order, data = rate_1990) else NULL

fit_rlm_2021 <- if (nrow(rate_2021) > 0) rlm(val ~ weighted_order, data = rate_2021) else NULL

fit_lm_1990 <- if (nrow(rate_1990) > 0) lm(val ~ weighted_order, data = rate_1990) else NULL

fit_lm_2021 <- if (nrow(rate_2021) > 0) lm(val ~ weighted_order, data = rate_2021) else NULL

coef_1990 <- if (!is.null(fit_rlm_1990)) coef(fit_rlm_1990) else c(NA, NA)

coef_2021 <- if (!is.null(fit_rlm_2021)) coef(fit_rlm_2021) else c(NA, NA)

pval_1990 <- if (!is.null(fit_lm_1990)) summary(fit_lm_1990)$coefficients["weighted_order", "Pr(>|t|)"] else NA

pval_2021 <- if (!is.null(fit_lm_2021)) summary(fit_lm_2021)$coefficients["weighted_order", "Pr(>|t|)"] else NA

write.csv(

data.frame(Year = c(1990, 2021),

Slope = c(coef_1990[2], coef_2021[2]),

P_Value = c(pval_1990, pval_2021)),

"regression_results_1990_2021.csv", row.names = FALSE

)

# ----------------------------- Plot: inequality scatter (Fig5A) ---

cols <- c("1990" = "#6699FF", "2021" = "#990000")

p1 <- ranked %>%

filter(metric_name == "Rate", year %in% c(1990, 2021)) %>%

mutate(year = factor(year)) %>%

ggplot(aes(x = weighted_order, y = val, color = year, fill = year)) +

geom_point(aes(size = pop/1e6), alpha = 0.8, shape = 21) +

scale_size_area("Population (million)", max_size = 10, breaks = c(100, 200, 400)) +

geom_smooth(method = "rlm", size = 0.6, alpha = 0.1, se = FALSE) +

scale_color_manual(values = cols) +

scale_fill_manual(values = cols) +

geom_segment(x = 0.02, xend = 0.99, y = coef_1990[1], yend = coef_1990[1],

color = cols["1990"], linetype = 2, linewidth = 0.4, alpha = 0.4) +

geom_segment(x = 0.02, xend = 0.99, y = coef_2021[1], yend = coef_2021[1],

color = cols["2021"], linetype = 2, linewidth = 0.4, alpha = 0.4) +

ggrepel::geom_text_repel(

data = subset(ranked, metric_name == "Rate" & location_name %in% c("China", "India") & year %in% c(1990, 2021)),

aes(label = location_name), size = 3, show.legend = FALSE

) +

annotate("text", label = paste0("Slope 1990: ", round(coef_1990[2], 2)),

x = 0.95, y = coef_1990[1], hjust = 1, vjust = -0.5, size = 3.5, color = cols["1990"]) +

annotate("text", label = paste0("Slope 2021: ", round(coef_2021[2], 2)),

x = 0.95, y = coef_2021[1], hjust = 1, vjust = -0.5, size = 3.5, color = cols["2021"]) +

scale_x_continuous(limits = c(0, 1), breaks = seq(0, 1, 0.1), labels = percent) +

labs(x = "Relative rank by SDI", y = "Crude DALY rate (per 100,000)",

title = "Global Health Inequalities: 1990 vs 2021") +

theme_bw()

ggsave("Fig5A_Inequality_Scatter.png", p1, width = 10, height = 8, dpi = 300)

ggsave("Fig5A_Inequality_Scatter.tiff", p1, width = 10, height = 8, dpi = 300, device = "tiff", compression = "lzw")

write.csv(

ranked %>% filter(metric_name == "Rate") %>% select(location_name, year, weighted_order, val, pop),

"Fig5A_Inequality_Scatter_Data.csv", row.names = FALSE

)

# ----------------------------- Concentration index (Number) -------

totals <- ranked %>%

filter(metric_name == "Number") %>%

group_by(year) %>%

summarise(total_daly = sum(val, na.rm = TRUE), .groups = "drop")

ci_df <- ranked %>%

filter(metric_name == "Number") %>%

arrange(year, sdi) %>%

group_by(year) %>%

mutate(cum_daly = cumsum(val),

frac_daly = cum_daly / totals$total_daly[match(year, totals$year)],

frac_population = cumsum(pop) / pop_global) %>%

ungroup()

temp1990 <- ci_df %>% filter(year == 1990)

temp2021 <- ci_df %>% filter(year == 2021)

CI_1990 <- 2 * mean(temp1990$frac_daly, na.rm = TRUE) - 1

CI_2021 <- 2 * mean(temp2021$frac_daly, na.rm = TRUE) - 1

# ----------------------------- Plot: Lorenz/concentration (Fig5B) -

color_eq <- "#CD853F"

p2 <- ci_df %>%

ggplot(aes(x = frac_population, y = frac_daly, color = factor(year), fill = factor(year))) +

geom_segment(x = 0, xend = 1, y = 0, yend = 0, color = "gray", linewidth = 1) +

geom_segment(x = 1, xend = 1, y = 0, yend = 1, color = "gray", linewidth = 1) +

geom_segment(x = 0, xend = 1, y = 0, yend = 1, color = color_eq, linewidth = 0.7) +

geom_point(aes(size = pop/1e6), alpha = 0.75, shape = 21) +

geom_smooth(method = "gam", formula = y ~ ns(x, knots = c(0.25, 0.5, 0.75)),

linewidth = 0.6, alpha = 0.1, se = TRUE) +

scale_color_manual(values = cols, name = "Year") +

scale_fill_manual(values = cols, name = "Year") +

scale_size_area("Population (million)", max_size = 10,

breaks = c(200, 400, 600, 800, 1000, 1200)) +

annotate("text", label = "Concentration Index", x = 0.75, y = 0.35, size = 5) +

annotate("text", label = paste0("1990: ", round(CI_1990, 2)), x = 0.75, y = 0.30, size = 4, color = cols["1990"]) +

annotate("text", label = paste0("2021: ", round(CI_2021, 2)), x = 0.75, y = 0.25, size = 4, color = cols["2021"]) +

labs(x = "Cumulative fraction of population ranked by SDI", y = "Cumulative fraction of DALY",

title = "Concentration Curve of DALYs by SDI: 1990 vs 2021") +

theme_bw()

ggsave("Fig5B_Concentration_Curve.png", p2, width = 10, height = 8, dpi = 300)

ggsave("Fig5B_Concentration_Curve.tiff", p2, width = 10, height = 8, dpi = 300, device = "tiff", compression = "lzw")

write.csv(

ci_df %>% select(location_name, year, frac_population, frac_daly, pop),

"Fig5B_Concentration_Curve_Data.csv", row.names = FALSE

)

# ----------------------------- Yearly robust slopes (1990–2021) ---

results <- lapply(1990:2021, function(yr) {

tmp <- ranked %>% filter(metric_name == "Rate", year == yr)

if (nrow(tmp) < 2) return(NULL)

fit_r <- rlm(val ~ weighted_order, data = tmp)

fit_l <- lm(val ~ weighted_order, data = tmp)

ci <- confint(fit_l)

data.frame(

year = yr,

intercept = coef(fit_r)[1],

slope = coef(fit_r)[2],

intercept_lwr = ci[1,1], intercept_upr = ci[1,2],

slope_lwr = ci[2,1], slope_upr = ci[2,2]

)

})

results <- bind_rows(results)

write.csv(results, "robust_regression_results_1990_2021.csv", row.names = FALSE)

p_slope <- ggplot(results, aes(x = year, y = slope)) +

geom_point(color = "#6699FF", size = 2) +

geom_smooth(method = "lm", se = TRUE, color = "#6699FF") +

labs(x = "Year", y = "Slope Index of Inequality (SII)",

title = "Trend of Slope Index of Inequality (1990–2021)") +

theme_minimal() +

theme(panel.grid.major = element_line(color = "gray90"),

panel.grid.minor = element_line(color = "gray95"))

ggsave("SII_Trend_1990_2021.png", p_slope, width = 10, height = 6, dpi = 300)

# ----------------------------- Bootstrap difference (optional) ----

set.seed(123)

bootstrap_ci_diff <- function(x, y, n = 10000) {

replicate(n, mean(sample(y, length(y), TRUE)) - mean(sample(x, length(x), TRUE)))

}

boot_diffs <- bootstrap_ci_diff(temp1990$frac_daly, temp2021$frac_daly, n = 10000)

boot_p <- mean(boot_diffs >= 0)

boot_ci <- quantile(boot_diffs, c(0.025, 0.975))

write.csv(data.frame(P_value = boot_p, CI_Lower = boot_ci[1], CI_Upper = boot_ci[2]),

"bootstrap_results_frac_daly_diff.csv", row.names = FALSE)

**7.NVL_DALY_Decomposition_ByLocation.R**

library(data.table)

library(dplyr)

library(tidyr)

library(ggplot2)

# -------------------- Inputs --------------------

# Expect:

# ./data/nvl_merged.csv (GBD outputs)

# ./data/GBD_population/ (folder of population CSVs)

# Writes:

# DALY_decomposition_by_location.csv

# DALY_decomposition_plot_by_location.tiff

burden <- fread("./data/nvl_merged.csv")

pop_files <- list.files("./data/GBD_population", full.names = TRUE)

population <- rbindlist(lapply(pop_files, fread), use.names = TRUE, fill = TRUE)

# -------------------- Preprocess --------------------

population <- population %>%

select(location_name, year, age_name, val) %>%

mutate(age_name = gsub(" years", "", age_name))

burden <- burden %>%

mutate(age_name = gsub(" years", "", age_name))

age_levels <- c("<5","5-9","10-14","15-19","20-24","25-29","30-34","35-39",

"40-44","45-49","50-54","55-59","60-64","65-69","70-74",

"75-79","80-84","85-89","90-94","95+")

locations <- c(

"Global","High-income Asia Pacific","High-income North America","Western Europe",

"Australasia","Andean Latin America","Tropical Latin America","Central Latin America",

"Southern Latin America","Caribbean","Central Europe","Eastern Europe","Central Asia",

"North Africa and Middle East","South Asia","Southeast Asia","East Asia","Oceania",

"Western Sub-Saharan Africa","Eastern Sub-Saharan Africa","Central Sub-Saharan Africa",

"Southern Sub-Saharan Africa","Middle SDI","Low SDI","High SDI","High-middle SDI","Low-middle SDI"

)

# -------------------- Decomposition --------------------

decomposition_one <- function(loc) {

pop_1990 <- population %>%

filter(location_name == loc, year == 1990) %>%

select(age_name, pop = val)

pop_2021 <- population %>%

filter(location_name == loc, year == 2021) %>%

select(age_name, pop = val)

r_1990 <- burden %>%

filter(location_name == loc, year == 1990,

measure_name == "DALYs (Disability-Adjusted Life Years)",

metric_name == "Rate", cause_name == "Near vision loss") %>%

select(age_name, rate = val)

r_2021 <- burden %>%

filter(location_name == loc, year == 2021,

measure_name == "DALYs (Disability-Adjusted Life Years)",

metric_name == "Rate", cause_name == "Near vision loss") %>%

select(age_name, rate = val)

df <- full_join(pop_1990, pop_2021, by = "age_name", suffix = c("_1990","_2021")) %>%

full_join(r_1990, by = "age_name") %>%

rename(rate_1990 = rate) %>%

full_join(r_2021, by = "age_name") %>%

rename(rate_2021 = rate) %>%

filter(age_name %in% age_levels)

if (nrow(df) == 0) {

return(data.frame(

location_name = loc,

overall_difference = NA_real_,

a_effect = NA_real_,

p_effect = NA_real_,

r_effect = NA_real_,

a_percent = NA_real_,

p_percent = NA_real_,

r_percent = NA_real_

))

}

df <- df %>%

mutate(across(c(pop_1990,pop_2021,rate_1990,rate_2021), as.numeric)) %>%

arrange(factor(age_name, levels = age_levels)) %>%

mutate(

rate_1990 = rate_1990 / 1e5,

rate_2021 = rate_2021 / 1e5

)

if (any(!is.finite(df$pop_1990)) || any(!is.finite(df$pop_2021)) ||

any(!is.finite(df$rate_1990)) || any(!is.finite(df$rate_2021))) {

return(data.frame(

location_name = loc,

overall_difference = NA_real_,

a_effect = NA_real_,

p_effect = NA_real_,

r_effect = NA_real_,

a_percent = NA_real_,

p_percent = NA_real_,

r_percent = NA_real_

))

}

P1990 <- sum(df$pop_1990, na.rm = TRUE)

P2021 <- sum(df$pop_2021, na.rm = TRUE)

if (P1990 <= 0 || P2021 <= 0) {

return(data.frame(

location_name = loc,

overall_difference = NA_real_,

a_effect = NA_real_,

p_effect = NA_real_,

r_effect = NA_real_,

a_percent = NA_real_,

p_percent = NA_real_,

r_percent = NA_real_

))

}

a1990 <- df$pop_1990 / P1990

a2021 <- df$pop_2021 / P2021

r1990 <- df$rate_1990

r2021 <- df$rate_2021

a_eff <- (sum(a2021 * P1990 * r1990) + sum(a2021 * P2021 * r2021))/3 +

(sum(a2021 * P1990 * r2021) + sum(a2021 * P2021 * r1990))/6 -

(sum(a1990 * P1990 * r1990) + sum(a1990 * P2021 * r2021))/3 -

(sum(a1990 * P1990 * r2021) + sum(a1990 * P2021 * r1990))/6

p_eff <- (sum(a1990 * P2021 * r1990) + sum(a2021 * P2021 * r2021))/3 +

(sum(a1990 * P2021 * r2021) + sum(a2021 * P2021 * r1990))/6 -

(sum(a1990 * P1990 * r1990) + sum(a2021 * P1990 * r2021))/3 -

(sum(a1990 * P1990 * r2021) + sum(a2021 * P1990 * r1990))/6

r_eff <- (sum(a1990 * P1990 * r2021) + sum(a2021 * P2021 * r2021))/3 +

(sum(a1990 * P2021 * r2021) + sum(a2021 * P1990 * r2021))/6 -

(sum(a1990 * P1990 * r1990) + sum(a2021 * P2021 * r1990))/3 -

(sum(a1990 * P2021 * r1990) + sum(a2021 * P1990 * r1990))/6

overall <- a_eff + p_eff + r_eff

if (!is.finite(overall) || overall == 0) {

ap <- pp <- rp <- NA_real_

} else {

ap <- 100 * a_eff / overall

pp <- 100 * p_eff / overall

rp <- 100 * r_eff / overall

}

data.frame(

location_name = loc,

overall_difference = round(overall, 3),

a_effect = round(a_eff, 3),

p_effect = round(p_eff, 3),

r_effect = round(r_eff, 3),

a_percent = round(ap, 2),

p_percent = round(pp, 2),

r_percent = round(rp, 2)

)

}

decomp <- bind_rows(lapply(locations, decomposition_one))

fwrite(decomp, "DALY_decomposition_by_location.csv")

# -------------------- Plot --------------------

plot_df <- decomp %>%

pivot_longer(c(a_effect, p_effect, r_effect),

names_to = "component", values_to = "value") %>%

mutate(location_name = factor(location_name, levels = rev(locations)),

component = factor(component, levels = c("a_effect","p_effect","r_effect"),

labels = c("Aging","Population","Epidemiological change")))

p <- ggplot(plot_df, aes(x = location_name, y = value, fill = component)) +

geom_col() +

geom_point(data = decomp,

aes(x = location_name, y = overall_difference, color = "Overall change"),

size = 2.5, shape = 21, fill = "black", inherit.aes = FALSE) +

scale_fill_manual(values = c("#800080","#008080","#FFD700"), name = "Component") +

scale_color_manual(values = c("Overall change" = "black"), name = NULL) +

coord_flip() +

labs(x = "Location", y = "Effect value", title = "Decomposition analysis by location") +

theme_bw() +

theme(legend.position = "bottom")

ggsave(

filename = "DALY_decomposition_plot_by_location.tiff",

plot = p,

width = 15, height = 10, units = "in",

dpi = 600, device = "tiff", compression = "lzw"

)

**8. NVL_Frontier_Bootstrap_LOESS.R**

library(data.table)

library(dplyr)

library(tidyr)

library(ggplot2)

library(ggrepel)

set.seed(123)

# ---------------- Inputs ----------------

# Expect:

# ./data/case.csv (GBD outputs with columns incl. location_name, year, age_name,

# cause_name, metric_name, sex_name, measure_name, val)

# ./data/SDI_2021.csv (columns: location_name, year, SDI)

case <- fread("./data/case.csv")

sdi <- fread("./data/SDI_2021.csv")

# ---------------- Filter & merge ----------------

df <- case %>%

filter(age_name == "Age-standardized",

cause_name == "Near vision loss",

metric_name == "Rate",

sex_name == "Both",

measure_name == "DALYs (Disability-Adjusted Life Years)") %>%

select(location_name, year, val) %>%

left_join(sdi, by = c("location_name","year")) %>%

rename(sdi = SDI) %>%

filter(is.finite(val), is.finite(sdi)) %>%

arrange(year, sdi, desc(val))

# ---------------- Bootstrap frontier ----------------

B <- 100

boot_list <- lapply(1:B, function(b) {

bs <- df[sample.int(nrow(df), nrow(df), replace = TRUE), ]

bs <- bs %>%

group_by(year) %>%

arrange(sdi, .by_group = TRUE) %>%

mutate(frontier = cummin(val)) %>%

ungroup()

bs %>% select(location_name, year, sdi, val, frontier)

})

boots <- bind_rows(boot_list)

summ <- boots %>%

group_by(location_name, year, sdi, val) %>%

summarise(frontier = mean(frontier, na.rm = TRUE), .groups = "drop") %>%

mutate(eff_diff = val - frontier)

# ---------------- Trend (1990→2021) ----------------

vals_1990 <- summ %>% filter(year == 1990) %>% select(location_name, val_1990 = val)

vals_2021 <- summ %>% filter(year == 2021) %>% select(location_name, val_2021 = val)

trend_df <- left_join(vals_2021, vals_1990, by = "location_name") %>%

mutate(trend = ifelse(val_2021 > val_1990, "Increase", "Decrease")) %>%

select(location_name, trend)

summ <- summ %>% left_join(trend_df, by = "location_name")

# ---------------- Plot A ----------------

plotA <- ggplot(summ, aes(x = sdi, y = val)) +

geom_point(aes(color = factor(year)), size = 1.8) +

scale_x_continuous(breaks = seq(0, 1, 0.2), limits = c(0, 1)) +

scale_y_reverse() +

stat_smooth(aes(x = sdi, y = frontier), method = "loess", se = FALSE,

span = 0.2, color = "black", fullrange = TRUE) +

labs(x = "SDI", y = "Age-standardized DALY rate per 100,000", color = "Year") +

theme_bw()

# ---------------- Plot B ----------------

summ_2021 <- summ %>% filter(year == 2021)

top15 <- summ_2021 %>% arrange(desc(eff_diff)) %>% slice_head(n = 15)

low_sdi <- summ_2021 %>% filter(sdi < 0.5) %>% arrange(eff_diff) %>% slice_head(n = 5)

high_sdi <- summ_2021 %>% filter(sdi > 0.85) %>% arrange(desc(eff_diff)) %>% slice_head(n = 5)

plotB <- ggplot(summ_2021, aes(x = sdi, y = val)) +

geom_point(aes(color = trend), size = 2.5) +

scale_color_manual(values = c("Increase" = "#d73027", "Decrease" = "#4575b4"),

name = "Trend (1990→2021)") +

scale_x_continuous(breaks = seq(0, 1, 0.2), limits = c(0, 1)) +

scale_y_reverse() +

stat_smooth(data = summ, aes(x = sdi, y = frontier),

method = "loess", se = FALSE, span = 0.2,

color = "black", fullrange = TRUE) +

ggrepel::geom_text_repel(data = top15, aes(label = location_name),

size = 2.5, fontface = "bold", max.overlaps = 160, color = "black") +

ggrepel::geom_text_repel(data = high_sdi, aes(label = location_name),

size = 2.5, fontface = "bold", max.overlaps = 160, color = "darkred") +

ggrepel::geom_text_repel(data = low_sdi, aes(label = location_name),

size = 2.5, fontface = "bold", max.overlaps = 160, color = "darkblue") +

labs(x = "SDI", y = "Age-standardized DALY rate per 100,000") +

theme_bw()

# ---------------- Exports ----------------

ggsave("Figure_A_SDI_vs_DALY.tiff", plotA, width = 8, height = 6, units = "in",

dpi = 300, device = "tiff", compression = "lzw")

ggsave("Figure_B_SDI_vs_DALY_annotated.tiff", plotB, width = 8, height = 6, units = "in",

dpi = 300, device = "tiff", compression = "lzw")

plot_data <- summ %>%

group_by(year) %>%

mutate(eff_rank = rank(-eff_diff, ties.method = "first")) %>%

ungroup() %>%

select(location_name, year, sdi, val, frontier, eff_diff, eff_rank, trend)

fwrite(summ, "frontier_bootstrap_summary.csv")

fwrite(plot_data, "plot_data.csv")

**8. NVL_BAPC_ASR_Projection_2035.R**

library(data.table)

library(dplyr)

library(tidyr)

library(ggplot2)

library(BAPC)

library(INLA)

# -------------------- Inputs --------------------

# Expect:

# ./data/nvl_merged.csv (GBD outputs)

# ./data/GBDpop1990_2100.RData (provides GBDpop1990_2100)

# Writes:

# Both_ASR_data.csv

# BOTH_ASR_projection.pdf

# BOTH_ASR_projection.tiff

set.seed(123)

df <- fread("./data/nvl_merged.csv")

load("./data/GBDpop1990_2100.RData") # object: GBDpop1990_2100

preyear <- 2035

years_obs <- 1990:2021

years_all <- 1990:preyear

dfx <- df %>%

filter(

measure_name == "DALYs (Disability-Adjusted Life Years)",

cause_name == "Near vision loss",

metric_name == "Number",

sex_name == "Both",

location_id == 1,

age_id %in% c(1, 6:20, 30, 31, 32, 235)

)

age_ids <- sort(unique(dfx$age_id))

# -------------------- Cases matrix (Number) --------------------

num_obs <- dfx %>%

select(year, age_id, val) %>%

complete(year = years_obs, age_id = age_ids, fill = list(val = 0)) %>%

arrange(year, age_id)

num_mat <- num_obs %>%

pivot_wider(names_from = age_id, values_from = val) %>%

arrange(year)

num_mat <- as.data.frame(num_mat[, -1]) # drop year column

pre_mat <- matrix(NA_real_, nrow = preyear - max(years_obs), ncol = ncol(num_mat))

colnames(pre_mat) <- colnames(num_mat)

apcNum <- rbind(num_mat, pre_mat)

rownames(apcNum) <- years_all

colnames(apcNum) <- as.character(age_ids)

# -------------------- Population matrix --------------------

pop_df <- GBDpop1990_2100 %>%

filter(

year %in% years_all,

age_id %in% age_ids,

location_id == unique(dfx$location_id),

sex_name == unique(dfx$sex_name)

) %>%

select(year, age_id, val) %>%

arrange(year, age_id)

pop_mat <- pop_df %>%

pivot_wider(names_from = age_id, values_from = val) %>%

arrange(year)

pop_mat <- as.data.frame(pop_mat[, -1]) # drop year column

colnames(pop_mat) <- colnames(apcNum)

# -------------------- Standard weights (data-driven) --------------------

# Mean age shares over observed years, aligned to columns of apcNum/pop_mat

std_tbl <- pop_df %>%

filter(year %in% years_obs) %>%

group_by(age_id) %>%

summarise(w = mean(val, na.rm = TRUE), .groups = "drop") %>%

right_join(tibble(age_id = as.integer(colnames(apcNum))), by = "age_id") %>%

mutate(w = ifelse(is.na(w), 0, w)) %>%

mutate(w = w / sum(w))

stdweight <- std_tbl$w

# -------------------- Build APC object --------------------

gloAPC <- APCList(apcNum, pop_mat, gf = 5, agelab = colnames(pop_mat))

np <- preyear - max(years_obs)

# -------------------- Fit BAPC (with robust fallback) --------------------

perform_BAPC <- function() {

tryCatch({

BAPC(

gloAPC,

predict = list(npredict = np, retro = TRUE),

verbose = FALSE,

secondDiff = FALSE,

model = list(

age = list(model = "rw2", prior = "loggamma", param = c(1, 0.00005)),

period = list(include = TRUE, model = "rw1", prior = "loggamma", param = c(1, 0.00005)),

cohort = list(include = TRUE, model = "rw2", prior = "loggamma", param = c(1, 0.00005)),

overdis = list(include = TRUE, model = "iid", prior = "loggamma", param = c(1, 0.005))

),

stdweight = stdweight

)

}, error = function(e) {

BAPC(

gloAPC,

predict = list(npredict = np, retro = TRUE),

verbose = FALSE,

secondDiff = FALSE,

stdweight = stdweight

)

})

}

glores <- perform_BAPC()

# -------------------- Age-standardized rate and CIs --------------------

asr <- agestd.rate(glores) %>% as_tibble() %>%

mutate(Time = years_all, group = "ASR") %>%

set_names("val", "sd", "Time", "group") %>%

mutate(val = val * 1e5, sd = sd * 1e5)

dfa <- asr %>%

mutate(

low_50 = val - 0.674 * sd, up_50 = val + 0.674 * sd,

low_60 = val - 0.841 * sd, up_60 = val + 0.841 * sd,

low_70 = val - 1.036 * sd, up_70 = val + 1.036 * sd,

low_80 = val - 1.282 * sd, up_80 = val + 1.282 * sd,

low_95 = val - 1.960 * sd, up_95 = val + 1.960 * sd

)

# -------------------- Plot --------------------

p1 <- ggplot(dfa, aes(Time, val)) +

geom_point(color = "black") +

geom_line(color = "#a696c8") +

geom_ribbon(aes(ymin = low_95, ymax = up_95), fill = "#6b6ecf", alpha = 0.2) +

geom_ribbon(aes(ymin = low_80, ymax = up_80), fill = "#9e9ac8", alpha = 0.2) +

geom_ribbon(aes(ymin = low_70, ymax = up_70), fill = "#bcbddc", alpha = 0.2) +

geom_ribbon(aes(ymin = low_60, ymax = up_60), fill = "#dadaeb", alpha = 0.2) +

geom_ribbon(aes(ymin = low_50, ymax = up_50), fill = "#efedf5", alpha = 0.2) +

geom_point(data = dfa %>% filter(Time > max(years_obs)), color = "red") +

geom_line(data = dfa %>% filter(Time > max(years_obs)), color = "red") +

geom_vline(xintercept = max(years_obs), linetype = "dashed", color = "grey50") +

scale_x_continuous(breaks = seq(min(years_all), max(years_all), by = 5)) +

labs(y = "Age-standardized rate per 100,000", x = "Year") +

theme_classic(base_size = 14) +

theme(legend.position = "none")

ggsave("BOTH_ASR_projection.pdf", p1, width = 16, height = 10, units = "in")

ggsave("BOTH_ASR_projection.tiff", p1, width = 16, height = 10, units = "in", dpi = 300)

# -------------------- Export data --------------------

dfa_out <- dfa %>% mutate(sex = "Both") %>% select(Time, val, sd, starts_with("low_"), starts_with("up_"), sex)

fwrite(dfa_out, "Both_ASR_data.csv")
