# Supplementary material for: Female predominance and socio-demographic inequalities in global near vision loss burden: projected trends and disparities from 1990 to 2035
Source: Front Public Health. 2025 Nov 3;13:1611433. doi: 10.3389/fpubh.2025.1611433 (PMC12620233; doi:10.3389/fpubh.2025.1611433)
Supplement: Supplementary file 3 [file Supplementary_file_2.docx]

Supplementary Material 1: Detailed Methods

**Data Sources and Preprocessing**

Data on near vision impairment were obtained from the Global Burden of Disease (GBD) 2021 dataset, accessed via the IHME GBD Results Tool (Global Health Data Exchange, GHDx) . We downloaded sexand age-specific prevalence counts and age-standardized rates (per 100,000) of near vision loss for all countries and years (1990–2021), along with the corresponding YLD (years lived with disability) counts and rates. The GBD data include 95% uncertainty intervals (UIs) for each estimate, derived from 1,000 posterior draws as per GBD methodology. The Socio-Demographic Index (SDI) for each country-year was also extracted from the GBD, where SDI is the geometric mean of total fertility, education, and income indices (scaled 0–1) . We grouped countries into five SDI quintiles for inequality analyses.

All age-specific data were processed in R (version 4.x) using packages such as tidyverse. We verified that age-standardized rates reported by GBD matched values we could compute by applying the GBD worldstandard population weights. When computing derived metrics, we propagated uncertainty by applying the Delta method or by using the stored GBD draws directly. For example, the female predominance parameter was defined as the ratio of female ASR to male ASR for each country-year; its 95% UI was computed by taking the ratio of the corresponding draws. To analyze temporal trends, we also extracted population counts by age/sex from the GBD population data for each country-year; these were used as offsets or denominators in regression models as needed.

Joinpoint Regression Analysis To characterize changes in trends over time, we performed joinpoint (segmented) regression on the log of the age-standardized rates. In a joinpoint model, the log-rate is modeled as a piecewise linear function of calendar year, with “joinpoints” (break years) where the slope may change . For example, a model with one joinpoint at year $\tau$ can be written as: $$ \ln(\text{ASR}t) = \beta_0 + \beta_1 t + \beta_2\, (t-\tau)+ + \varepsilon_t,\qquad (t= \text{year}), $$ where $(t-\tau)_+ = \max{0,\,t-\tau}$ . Here $\beta_1$ is the slope before $\tau$, and $\beta_1+\beta_2$ is the slope after $\tau$. In general, with $k$ joinpoints the model has $k+1$ linear segments. We fitted these models using the segmented R package (v1.8-0) by first fitting a baseline linear model and then using segmented() to estimate the breakpoint(s).

Trend estimation and EAPC. To quantify time trends, we modeled the log of age-standardized rates (ASR) of near vision loss as a function of year. Joinpoint (segmented) regression models were fitted in R using the segmented package (Muggeo 2008) , allowing the slope to change at one or more breakpoints. The number of joinpoints was chosen using BIC/permutation tests. For each segment we computed the Annual Percent Change (APC) as $100\times(e^{\beta}-1)$ from the estimated linear slope β. We also computed an overall (average) EAPC for 1990–2021 by fitting a single linear model on log(val) versus year (using lm in R) and transforming the slope (and its CI) to percent change as above. In practice we implemented this by grouping data by country/age/cause, fitting lm(log(val)~year) , and using the broom package to extract slopes and 95% confidence intervals. Finally, point estimates and uncertainty (in percent) were output in the format “β (lower, upper)”.

**Frontier Analysis**

We evaluated each country’s efficiency gap relative to the empirical “frontier” of lowest NVL burden at its development level. We regressed the age-standardized DALY rate (response) on SDI (predictor) to estimate the best-achievable minimum. Instead of directly fitting a model, we used a nonparametric bootstrap-cum-minimum approach: we resampled the country-level data (with replacement) $B=100$ times (seed set for reproducibility) and, within each bootstrap sample and year, sorted countries by SDI and computed the cumulative minimum of the DALY rate. The resulting “frontier” for each bootstrap replicate is essentially the lowest-rate envelope by SDI. We then averaged these bootstrapped frontiers over replicates to obtain a smoothed frontier curve. Finally, we fitted a LOESS curve (using R’s stat_smooth(span=0.2) ) through the averaged frontier estimates across the SDI range. Bootstrap-based pointwise 95% uncertainty bands were obtained from the 2.5th and 97.5th percentiles of the predicted frontiers. For each country-year, the efficiency gap was defined as the difference (observed ASR minus frontier ASR at that SDI): a positive gap indicates burden above the frontier. (All computations used dplyr and base R functions; plotting was via ggplot2.)

**Decomposition Analysis**

To disentangle the drivers of change in near-vision DALY burden between 1990 and 2021, we applied a Das Gupta-style demographic decomposition . We used age-specific population counts ($N_{a,1990}$, $N_{a,2021}$) and age-specific DALY rates ($r_{a,1990}, r_{a,2021}$) for each location. Denote total burden $B_t=\sum_a N_{a,t}r_{a,t}$. Then the change $B_{2021}-B_{1990}$ was partitioned into three additive components: population growth, population aging (structure change), and epidemiological change (rate change). In practice we implemented the exact Das Gupta formula (continuous change model) in R: computing contributions of aging, growth, and rate effects for each age group and summing.

(Our code uses closed-form expressions equivalent to the symmetric Das Gupta decomposition .) For each location (global, regions, superregions, and SDI groups), the raw contributions (Δ by component) and their percent shares of the total change were calculated. These were reported in the supplement as absolute and percentage contributions

**Bayesian Age-Period-Cohort (BAPC) Modeling**

For projections to 2035, we used a Bayesian age-period-cohort model as implemented in the R BAPC package . This model assumes count data $Y_{a,p}$ (e.g. cases or YLD) for age group $a$ and period $p$ follow a Poisson distribution with mean $\mu_{a,p} = N_{a,p}\lambda_{a,p}$, where $N_{a,p}$ is the population and $\lambda_{a,p}$ the rate. The log-rate is modeled additively as $$ \log(\lambda_{a,p}) = \mu + \alpha_a + \beta_p + \gamma_c, $$ where $\alpha_a$, $\beta_p$, and $\gamma_c$ are the (random) age, period, and cohort effects, and $c=p-a$ is the birth cohort. BAPC places second-order random walk priors on these effects to induce smoothness, and fits the model using Integrated Nested Laplace Approximation (INLA) . This approach handles over-dispersion and provides full posterior intervals with relatively fast computation.

The BAPC object contains posterior draws of age-specific and age-standardized rates for each projected year, from which we extracted point estimates and 95% credible intervals. We verified model fit by inspecting the posterior predictive distributions and checking that the historical data were well-reproduced. We also ran sensitivity tests with different prior hyperparameters (using bapc_fit$hyperpars adjustments) and confirmed stability of projections.

Finally, to obtain overall projections for aggregated groups (e.g. sexes or SDI quintiles), we combined the age-specific projections using population weights. For example, the projected ASR for females in 2035 was computed as the sum of the projected female age-specific rates times the 2035 female standardpopulation weights. We report these sex-specific and SDI-specific projections (with 95% credible intervals) in the revised Results section.

References to Software and Equations: All R code was executed in R 4.2.3 or later. Besides segmented, loess, DemoDecomp, and BAPC, we used dplyr/tidyverse for data manipulation and ggplot2 for plotting. Where relevant, we have cited the original methodology (e.g. Muggeo 2008 for segmented regression , Riebler 2020 for BAPC ) and GBD technical notes. We trust that this Supplementary Material provides the complete methodological detail requested by the reviewer.
